# Improved *Glycine max* productivity in saline–sodic soils: coupling the impacts of urea–phosphate and magnesium oxide nanoparticles on the nutrient contents and growth–physiological attributes

**DOI:** 10.1186/s12870-025-07445-2

**Published:** 2025-10-21

**Authors:** Safaa A. Abou-Zaid, Mohamady I. El-Kherbawy, Ahmed A.M. Awad, Atef A.A. Sweed

**Affiliations:** 1 Soil and Natural Resources Department, Faculty of Agriculture & Natural Resources, Aswan University, Aswan, 81528 Egypt; 2 Soil and Water Department, Faculty of Agriculture, Cairo University, PO Box 12613, Giza, Egypt

**Keywords:** Phosphorus fertilizer, Magnesium oxide nanoparticles, Salt-affected soils, Leaves’ nutrient contents, Soybean plant

## Abstract

**Background:**

Among abiotic stresses, salinity and socidity are the major productivity-limiting factors for cultivated crop plants. In two seasons (2022 and 2023), two field-level experimental attempts were made to study the impacts of urea–phosphate (UP) and magnesium oxide nanoparticles (MgNPs) and their combination on the nutritional status, physiological attributes, and yields of *Glycine max* (L) plants growing under saline-sodic conditions. At 30, 45, and 60 days, UP was applied to the soil at rates of 85.0, 107.0, 127.0, and 150.0 kg ha^-1^, corresponding to UP_1_, UP_2_, UP_3_, and UP_4_, respectively, as well as MgONPs via foliar application at doses of 0.0, 50.0, and 100.0 mg L^-1^, corresponding to MgONP_0_, MgONP_1_, and MgONP_2_, respectively. This study was conducted in a split-plot structure according to a randomized compete block design with three replicates.

**Results:**

Our results showed that UP_3_ and UP_4_ had the strongest effects on most of the measured traits. Both application rates produced the maximum growth–physiological attribute values, except for the leaf dry matter percentage and leaf nutrient levels in both seasons and the leaf iron and zinc contents in the first season (the highest values in these characteristics were achieved in the untreated plants). In addition, the UP_4_-fertilized plants produced the highest 100-seed weight (HSW), total seed yield (TSY), and seed manganese contents in the two growing seasons. The highest seed oil contents in both seasons, as well as the highest seed phosphorus, calcium, and copper contents in 2023, were recorded in the UP_3_-treated plants. Regarding MgONPs, our results revealed the significant superiority of the MgONP-treated plants in terms of all the aforementioned growth–physiological parameters and leaf macro- and micronutrient contents, irrespective of the applied dose, except for the LMnCs in 2022 and 2023. The integrative application revealed the clearly superior influence of applying UP_3_ or UP _4_ with MgONP _2_ and MgONP _3_ on most studied traits. Specifically, for TSY, Model 3 in the 2022 season (adjusted *R*^*2*^_*2*_ = 0.931) and Model 2 in LPC, LA, and LCaC as the most influential attributes in 2022, and LNC and LKC in 2023. For seed oil content (SOC), Model 3 in 2022 (adjusted *R*^*2*^_*2*_ = 0.344) and Model 2 in 2023 (adjusted *R*^*2*^_*2*_ = 0.278) were selected, with SPAD readings and LMnC in 2022, and LMnC and HSW in 2023 as key predictors.

**Conclusions:**

The use of high rates of a highly soluble and acidic-impact fertilizer, such as UP supplemented with magnesium oxide nanoparticles, may be recommended in saline environments.

## Introduction

Egypt, like other developing countries, suffers from extreme population growth, which has resulted in food insecurity [1]. Globally, it is expected that the world’s population will exceed 9.7 billion by 2050, which will require increasing food production by over 70% [2]. Achieving self-sufficiency is one of the most important pillars for achieving food security, including producing edible oil in sufficient amounts. Currently, there is a gap between food production and consumption, which is exacerbated daily due to increasing population growth and limited agricultural land [3]. Egypt produces less than 10% of the food that it consumes; accordingly, it imports approximately 90% of the oil required for its total oil consumption. In addition, Egypt’s arable lands are being degraded due to their gradual conversion to salt-affected soils, which negatively affects the growth and development of plants through its influence on their physiological and metabolic processes and, ultimately, crop productivity [4]. In this regard, soil salinity as a result of its detrimental impacts on agricultural productivity, mainly in arid and semi-arid regions is the most significant global agricultural threat that challenges the objectives of sustainable development [5]. This phenomenon occurs in more than 100 countries worldwide and is defined by the localized accumulation of soluble salts around root zones. Soil salinity results from two main factors: natural causes (primary salinization) and anthropogenic activities (secondary salinization) [6]. According to projections, approximately 6% of the world’s land will be subjected to soil salinity, accounting for more than 800 million hectares [7],, which is estimated to impact 50% of the world’s total land by 2050 [8]. To address this challenge, attention must be paid to removing the obstacles to improving soil functions. Improved soil is associated with crop productivity and cultivation, including oil crops, of which soybean (*Glycine max* L. Merr) is considered the most significant based on its nutritional value [9]. Soybean is the most essential and important annual legume due to its multiple uses for human food and livestock feed worldwide [10–12], and its edible oil is an essential source of high-quality proteins and is rich in valuable amino acids, such as lysine (which is low in most grain crops), as well as vitamins, niacin, riboflavin, and micronutrients [13]. In addition, its seeds contain about 20–22% oil, 30–35% carbohydrates, 10–12% sugars, and 42–45% protein [14]. According to [15], the total cultivated soybean area in Egypt has reached about 11,900 hectares, and its total production has reached about 35,000 tons, with an average of 2.94 tons per hectare. Meanwhile, the worldwide cultivated soybean area has reached 121.5 million ha, with a total yield of about 334.9 million tons.

Several strategies have been adopted to overcome the harmful impact of salinity on crops. Among them is balanced nutrition, which depends mainly on phosphorus (P) and magnesium (Mg) for maximizing and optimizing yields. Thus, these two nutrients are among the most crucial and influential for soybean plants. P plays a significant role in root development, resulting in improved nutrient and water uptake and enhanced reproduction, flowering, pod setting, and seed formation, which boost crop productivity [16–18]. In addition, P plays a pivotal role in several metabolic processes, including protein synthesis, sugar translocation, photosynthesis, respiration, building new tissues, cell division, and elongation, and it is an integrated constituent in nucleic acids, energy-rich compounds, synthesis phospholipids, and N fixation [19]. Similarly, Mg is one of the most important nutrients to the productivity of crops, playing a significant role in many physiological and metabolic processes in plants, such as photosynthesis, photoprotection, and carbohydrate synthesis [20, 21]. In addition, Mg activates several enzymes, such as glutathione synthase, RNA polymerase, phosphoenolpyruvate carboxylase, ATPases, and all enzymes linked to the photosynthesis process [22]. Many reviews have been published on the importance of Mg to the photosynthetic performance, in the formation of roots and shoots, and in improving cellular stress defense mechanisms [23–25]. Various factors may lead to Mg deficiency in plants, such as high pH, low Mg concentrations owing to CaCO_3_ contents, and the presence of other nutrients that fix Mg in its unavailable form [26]. The same authors noted that in soils with adequate Mg contents, Mg deficiency in plants may nevertheless appear due to soil salinity, low water availability, and low plant transpiration. Furthermore, high soil Mg levels may hinder zinc and manganese absorption, causing deficiencies in both nutrients [27]. Therefore, in recent decades, the use of nanofertilizers (NFs) has gained a great deal of attention due to their distinctive properties [28]. At present, the application of NFs is considered one of the most effective methods in agricultural systems [29, 30] because of their unique properties, including their large specific surface areas that allow for gradual release, small diameters of less than 100 nm, low weights, and electronic states [31, 32]. There are various specific strategies for producing NFs, including chemical and physical processes as conventional methods and green synthesis, which has gained paramount attention due to its environmentally friendly nature [33]. Numerous studies have reported that NFs enhance plant resistance against abiotic and biotic stresses [34], in addition to improving plant growth and crop production [35–37]. Among the various inorganic metal oxides, magnesium oxide nanoparticles (MgONPs) are being increasingly used as an antimicrobial agent [38]. Numerous studies have applied MgONPs to enhance plant growth and crop productivity [39, 40]. It is worth noting that plants uptake only 10–20% of the P applied to the soil, while the remainder often percolates into subsurface layers, leading to soil and water pollution Consequently, applying excessive amounts of P is not recommended for maintaining soil fertility [41–43]. Accordingly, this research was conducted to assess the potential impacts of the individual use of four UP levels as a soil application and three MgONPs doses as a foliar spray, as well as their interactive effects on leaf nutrient contents, growth-physiological attributes, and productivity of soybean grown under sandy saline-sodic conditions. Under these conditions, soil P is largely immobilized and bound into less soluble Mg and Ca compounds.

## Materials and methods

### Overview of field study location, experimental details, and weather conditions

The two seasons of 2022 and 2023 were specified to perform two field experiments at the experimental farm at the Faculty of Agriculture and Natural Resources, Aswan University campus (24° 05` 20"N and 32° 53’ 59"E), Egypt. This study was conducted to investigate the individual influence of urea phosphate (UP) and magnesium oxide nanoparticles (MgONPs) and the influence of their interaction on the nutritional status, yield, and components of soybean (*Glycine max* L. Giza 111 cv.) plants cultivated under a drip irrigation system in saline soils. This selected cultivar was obtained from the Oil Crops Research Department, Agricultural Research Center (ARC) in Giza, Egypt. This cultivar was chosen for its high adaptability to local conditions, prompt germination, and superior productivity. The average weather information (from April to August) in both growing seasons of the study location is presented in Table 1.

**Table 1:** The average Climatic information of the study location (24° 05` 20"N and 32° 53’ 59"E) during the soybean growing seasons (2021 and 2022)

Month	ADT	ANT	ARH	ASW	AM-PEC-A	AP
	(ºC)		(%)	(m s^−1^)	(mm d^−1^)	
2021 growing season						
April	29.15	26.82	80.69	4.34	8.68	2.50
May	29.19	27.15	77.25	4.12	7.18	0.49
June	30.22	27.08	77.88	4.31	6.39	2.08
July	29.33	25.62	81.38	5.04	5.48	3.11
August	28.97	26.70	78.88	4.41	4.81	0.83
2022 growing season						
April	29.23	26.04	81.19	3.17	9.46	10.28
May	29.79	26.90	81.38	3.54	8.92	7.45
June	29.70	26.92	79.25	3.57	6.82	4.48
July	29.95	26.94	81.31	3.71	5.94	4.09
August	29.55	27.24	78.56	4.16	4.83	3.96

ADT and ANT — Average of day and night temperatures, respectively. ARH — average of relatively humid, AMP-A — average of measured pan evaporation Class A. ASW — average of wind speed, and AP — average precipitation. https://power.larc.nasa.gov/index.php

### Intercultural operations, treatment, and experimental layout

Soybean seeds were sown on April 14, 2022, and April 23, 2023, respectively. Four rates of UP were applied to the soil (85.0, 107.0, 127.0, and 150.0 kg ha^−1^ of UP_1_, UP_2_, UP_3_, and UP_4_, respectively) as a main factor, and three doses of MgONP were used as a foliar spray (0.0, 50.0, and 100.0 mg L^−1^; MgNP_0_, MgNP_1_, and MgNP_2_, respectively) as a submain factor. The main experimental units were arranged according to the four levels of UP. Each main unit was subsequently subdivided into three treatments of MgONP. Accordingly, there were 4 × 3 = 12 treatment interactions. The area of each main plot was 10.6 m (length) x 6.0 m (width) = (63.0 m^2^). The soybean plants were treated with UP in a solid form and with foliar-applied MgONP solutions three times (45, 60, and 75 days after sowing (DAS) using a 10 L back sprayer. The recommended fertilization program depended on applying ammonium sulfate [(NH_4_)_2_SO_4_ N%≈20.6)] as a nitrogen source, with 192 kg N ha^−1^ in four equal doses at a rate of 48 kg N ha^−1^ during the first four irrigations, and applying potassium sulfate [K_2_SO_4_ K_2_O ≈ 48–52%] as a K-source, at a rate of 125 kg K_2_SO_4_ ha^−1^, before the second irrigation. The UP (NH_2_)_2_-CO.H_3_PO_4_ ≈ 17.72–44.72-0.72) was applied as a phosphorus fertilizer, and its level was calculated as P_2_O_5_% based on its P content. Three seeds were sown in each hill (at a rate of 3–4 per hill), with a distance of 15 cm between hills. The total amount of seeds was 75 kg ha^−1^.

Characterization of MgONPs.

**Fig. 1:**
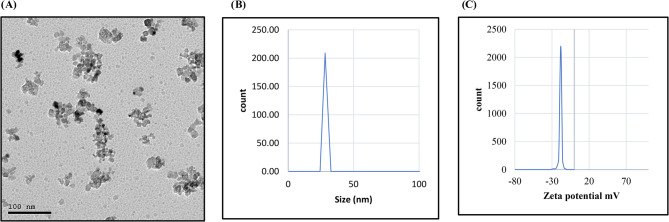
Transmission electron micrograph (TEM) image (**A**), particle size distribution (PSD) curve (**B**), and zeta potential analysis (**C**) of magnesium oxide nanoparticles (MgONPs)

The MgONPs were purchased from Sigma-Aldrich, St. Louis, Mo, USA. The size of each subplot was 3.5 m (length) x 2.0 m (width) = (7.0 m^2^). All intercultural operations were carried out according to technical bulletin no. 1412 issued in 2022 by the ARC. The compositions of all the applied treatments are shown in Table 2. The results obtained from transmission electron microscopy (TEM) of the MgONPs indicated that the particles were considerably spherical, dispersed, and uneven in nature, and their average sizes were 100 nm, as shown in Fig. 1A. The MgONP particle size distribution (PSD) was determined via dynamic light scattering (DLS) using a particle size analyzer, and it ranged between 0 and 50 nm, as presented in Fig. 1B.

**Table 2:** The details of applied experiment treatments

UP	MgONPs	Composition treatment	Replication and applying time	Symbol
UP_1_	MgONP_0_	85.0 kg of UP and no MgONPs was applied	The UP treatments were applied three timed after 45, 60 and 75 days after sowing (DAS). The MgONPs solutions were foliar-applied three times in three replicates	T_10_
	MgONP_1_	85.0 kg of UP and + 50.0 mg L^−1^ of MgONPs was applied		T_11_
	MgONP_2_	85.0 kg of UP and + 100.0 mg L^−1^ of MgONPs was applied		T_12_
UP_2_	MgONP_0_	107.0 kg of UP and no MgONPs was applied		T_20_
	MgONP_1_	107.0 kg of UP and + 50.0 mg L^−1^ of MgONPs was applied		T_21_
	MgONP_2_	107.0 kg of UP and + 100.0 mg L^−1^ of MgONPs was applied		T_22_
UP_3_	MgONP_0_	127.0 kg of UP and no MgONPs was applied		T_30_
	MgONP_1_	127.0 kg of UP and + 50.0 mg L^−1^ of MgONPs was applied		T_31_
	MgONP_2_	127.0 kg of UP and + 100.0 mg L^−1^ of MgONPs was applied		T_32_
UP_4_	MgONP_0_	150.0 kg of UP and no MgONPs was applied		T_40_
	MgONP_1_	150.0 kg of UP and + 50.0 mg L^−1^ of MgONPs was applied		T_41_
	MgONP_2_	150.0 kg of UP and + 100.0 mg L^−1^ of MgONPs was applied		T_42_

UP urea-phosphate. MgONPs magnesium oxide nanoparticles

### Soil sample collection and evaluation of soil properties

Before sowing, 30 soil samples were randomly collected from the surface soil layer (0–30 cm) and were then mixed into a composite sample to determine various chemical and physical properties (Table 3). Based on the values obtained for the sodium adsorption ratio (SAR) and exchangeable sodium percentage (ESP), the studied soils were classified as saline–sodic soil according to the USDA, with an ECe > 4 dS m^−1^, a pH < 8.5, and an SAR > 15.

**Table 3:** Some chemical and physical properties of the experimental farms in 2022 and 2023

Soil properties	Unit	Reference	Value	
			2022	2023
Particle size distribution				
Sand	(%)	[44]	89.40	88.10
Silt			4.70	4.50
Clay			5.80	5.90
Soil texture			Sandy	Sandy
pH in soil paste		[45]	7.56	7.68
ECe in soil paste extract	(dS m^−1^)	[46]	7.89	8.32
OM	(%)	[47]	0.68	0.89
CaCO_3_		[46]	4.25	4.37
Soluble ions (cations and anions)				
Na^+^	(meq L^−1^)	[47]	57.00	59.00
K^+^			3.50	4.00
Ca^2+^			8.50	8.00
Mg^2+^			6.00	5.50
CO_3_^2−^	(meq L^−1^)	[45]	0.00	0.00
HCO_3_^−^			6.10	5.50
Cl^−^			57.40	59.00
SO_4_^2−^			11.50	1200
SAR			15.00	16.07
ESP	**(%)**		16.66	17.55
Available macronutrients				
N	(mg kg^−1^)	[48]	160.00	188.00
P-Extractable with NaHCO_3_ pH 8.5		[49]	1332.00	1522.00
K-Extractable with NH_4_AC pH 7.0		[50]	157.00	178.00
Available micronutrients				
Fe-Extractable with DTPA	(mg kg^−1^)	[51]	36.30	35.40
Mn- Extractable with DTPA			17.60	16.20
Zn- Extractable with DTPA			12.60	11.80
Cu- Extractable with DTPA			4.80	3.20

EC-electrical conductivity. OM-organic matter. SAR-sodium adsorption ratio. And ESP-Exchangeable sodium percentage

### Growth–physiological parameters

After 90 days had elapsed since sowing, as a representative sample of those sown, five plants were collected from each experimental unit to determine the growth–physiological attributes. The relative chlorophyll content (SPAD reading) was measured using the SPAD-502 (Minolta Sensing, Inc., Sakai, Osaka, Japan). The plant height (PH) was measured above the soil surface using a meter scale (in centimeters), while leaf area (LA) was measured using a planimeter device. The plant dry matter percentage (%PDrM) was determined based on fresh weight (PFrW) and dry matter (PDrM). To determine the latter, the plants were dried in an oven at 72 °C for 48 h, and PDrM was calculated according to the following equation: %PDrM=$\:\frac{\left(\text{P}\text{F}\text{r}\text{W}\right)-\left(\text{P}\text{D}\text{r}\text{M}\right)}{\left(\text{P}\text{D}\text{r}\text{M}\right)}\:100$.

### Determination of leaf macro- and micronutrient content

According to the methods described in [52], the plant nitrogen (LNC), phosphorus (LPC), potassium (LKC), calcium (LCaC), and magnesium (LMgC) were analyzed in the plant dry tissue using 0.2 g of leaf dry matter digested with sulfuric and perchloric acids. Leaf micronutrient contents, such as iron (LFeC), manganese (LMnC), zinc (LZnC), and copper (LCuC) content, were determined using an inductively coupled plasma optical emission spectrometer (ICP-OES, Perkin-Elmer OPTIMA-2100DV, Norwalk, CT, USA) according to the method described in [51].

### Statistical analysis of the resulting data

In both seasons, analysis of variance (ANOVA) of the split-plot structure was conducted according to a randomized complete block design using the Infostat statistical package, version 9.2 (INFOSTAT, 2019), according to the procedures outlined in [53]. Pearson correlation coefficients were applied to determine the relationship between the studied attributes, and stepwise regression was calculated to explain the relationship between the TSY and related parameters using IBM SPSS statistics 2021 Wizard.

## Results

### Growth and physiological attributes

#### Response of growth–physiological attributes in salt-stressed soybean plants to UP soil application

**Fig. 2:**
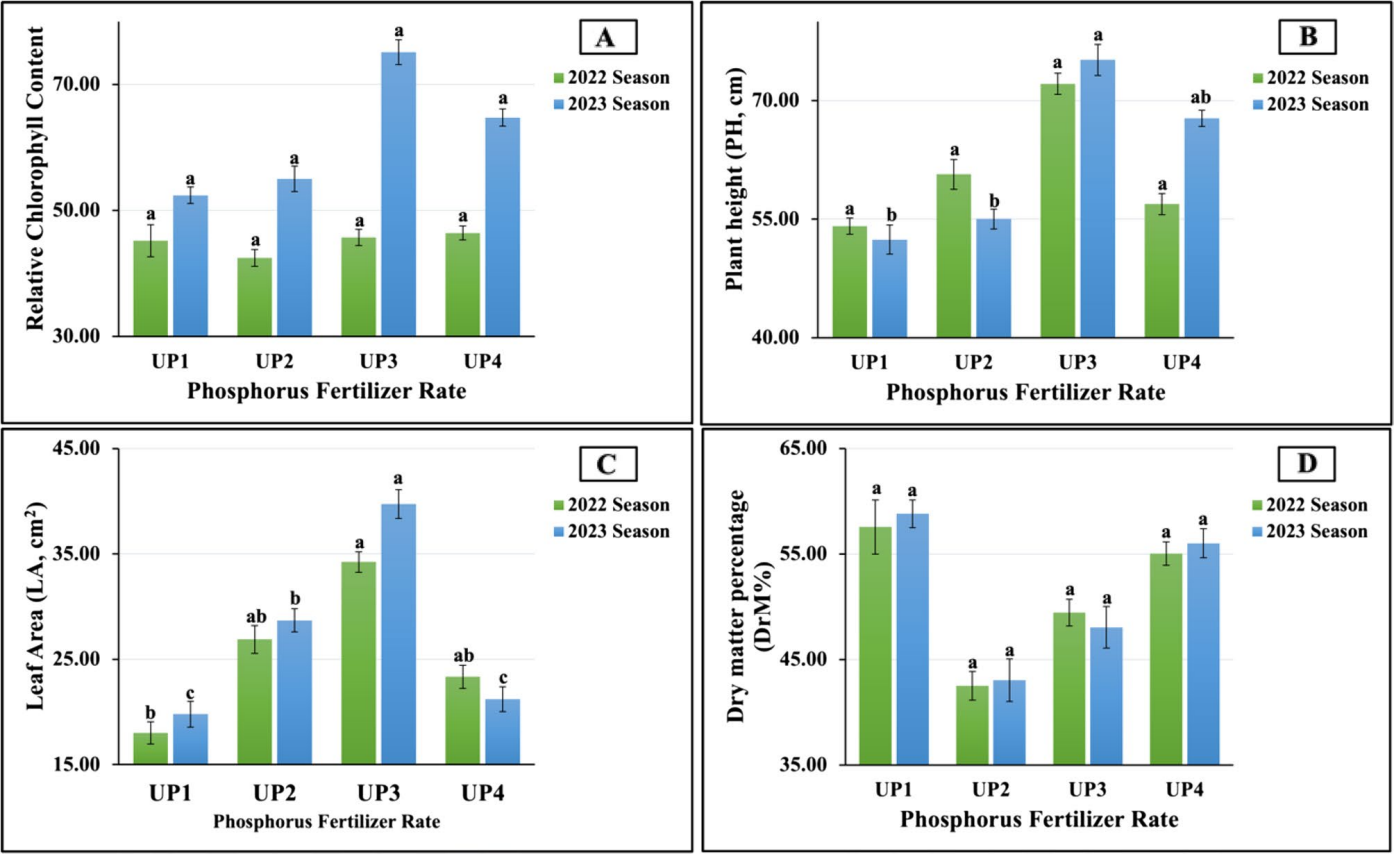
**A**-**D** The individual effect of urea-phosphate fertilizer rates (UPs) on 1 A) SPAD reading, 1B) Plant height, 1 C) leaf area, and 1D) leaf dry matter percentage (DrM%) of soybean plants cultivated in saline soil in both growth seasons 2022 and 2023, respectively. UP_1_, UP_2_, UP_3_, and UP_4_ represent urea-phosphate at 85.0, 107.0, 127, and 150.0 kg ha^−1^, respectively. The data are means±SE (Standard Error) for three replicates. Means value that have different lower-case letter in each season are significant at *p* ≤ 0.05 according to Duncan’s multiple range test

The data pertaining to the effect of urea phosphorus (UP) fertilizer rates on growth and physiological parameters, such as relative chlorophyll content (SPAD reading), plant height (PH), leaf area (LA), and plant dry matter percentage (DrM%), of salt-stressed soybean plants in the 2022 and 2023 growing seasons are graphically presented in Fig. 2 (A-D). The results obtained indicate that the maximum values in PH (72.11 vs. 75.14 cm) and LA (34.22 vs. 39.72 cm^2^) in both growth seasons were recorded in plants fertilized with UP_3_; moreover, the maximum value in SPAD (53.67) was recorded in the second season. Meanwhile, the application of the UP_1_ significantly increased DM% compared to higher UP levels. So, the highest values in DrM% were recorded in both growing seasons (57.55 vs. 58.80% in 2022 and 2023, respectively). On the other hand, UP_1_ was the least influential variable on PH (54.11 vs. 52.41 cm) and LA (18.01 vs. 19.79 cm²) in the first and second seasons and the least influential on SPAD readings (43.67) in the second season. The lowest DrM% values (42.50 vs. 43.04%) in both seasons and the lowest SPAD reading (42.47) in the first season were produced by plants treated with UP_2_. The analysis of variance presented a significant effect (at *p* ≤ 0.01) on LA in the 2023 season and a significant impact (at *p* ≤ 0.05) on PH in the second season and on LA in the first season. In addition, a non-significant influence was demonstrated by the SPAD reading and DrM% in both growing seasons, as well as by pH in the first season.

#### Response of leaf nutrient contents in salt-stressed soybean plants to mgonp foliar application

**Fig. 3:**
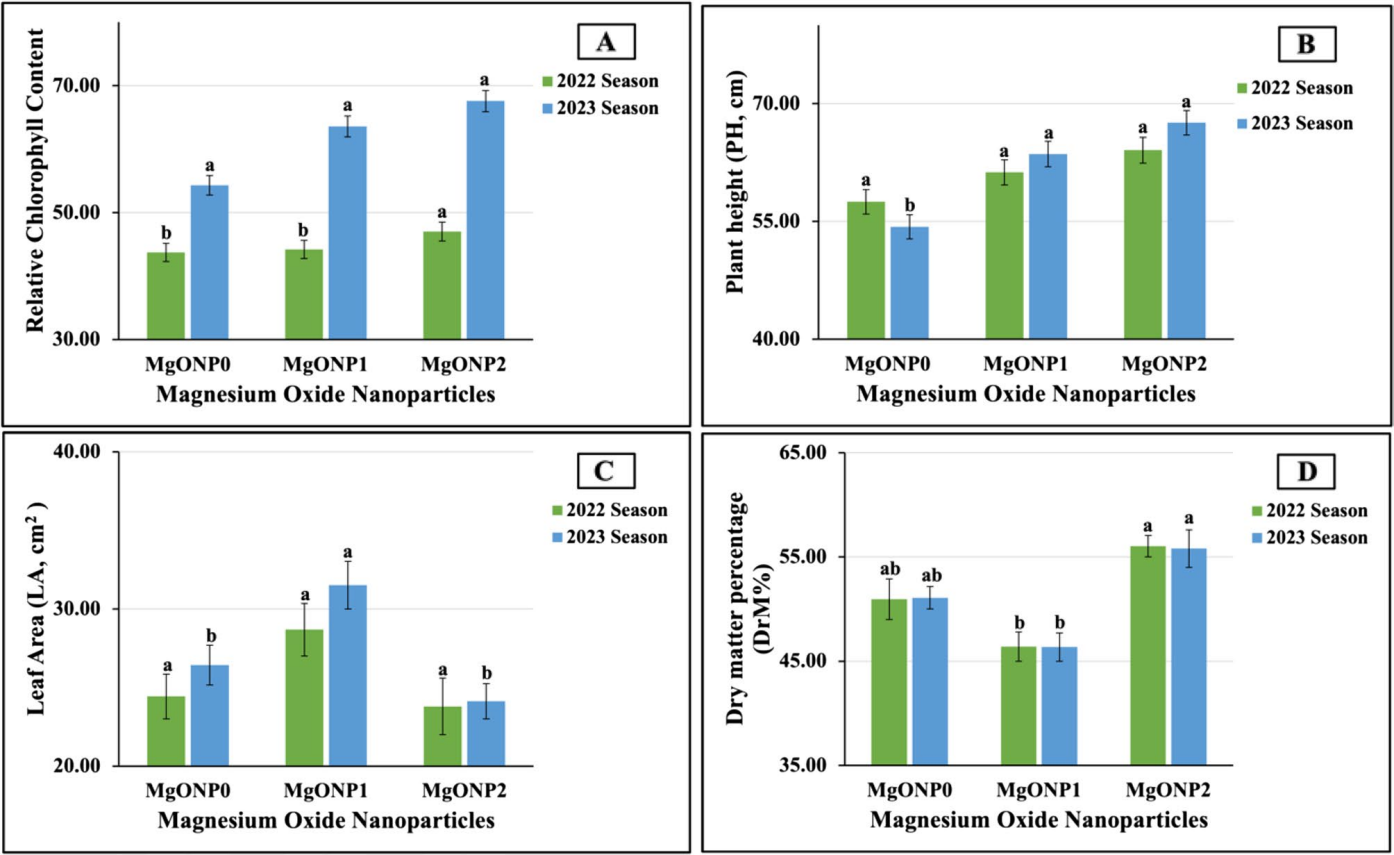
**A**-**D** The individual effect of magnesium oxide nanoparticle doses (MgONPs) on (**A**) SPAD reading, **B** Plant height, **C** leaf area, and **D** dry matter percentage of soybean plants cultivated in saline soil in both growth seasons 2022 and 2023, respectively. The data are means ± SE (Standard Error) for three replicates. Means value that have different lower-case letter in each season are significant at *p* ≤ 0.05 according to Duncan’s multiple range test

Figure 3 (A-D) graphically display the maximum values recorded in the leaves treated with MgONP_2_ in terms of SPAD readings (47.00 vs. 51.00 in 2022 and 2023, respectively), PH (64.08 vs. 67.57 cm in 2022 and 2023, respectively), and %DrM (56.63 vs. 55.81% in 2022 and 2023, respectively) in the first and second seasons. The highest LA values were achieved in the plants sprayed with MgONP_1_ (28.68 vs. 31.51 cm² in the 2022 and 2023 growing seasons, respectively). In contrast, the minimum values in terms of the SPAD readings (43.72 vs. 47.25 in 2022 and 2023, respectively) and PH (57.50 vs. 54.32 cm in 2022 and 2023, respectively) were produced in the untreated plants (MONP_0_) in both seasons. In addition, the lowest LA (23.80 vs. 24.12 cm² in 2022 and 2023, respectively) and DrM% (46.40 vs. 46.35% in 2022 and 2023, respectively) values in the 2022 and 2023 growth seasons were obtained in the plants treated with MgONP_2_ and MgONP_1_, respectively. Statistically, highly significant increases were recorded in the SPAD readings in 2022 and in the PH and LA values in 2023 following all the treatments. These treatments had no significant impacts on the PH or LA values in the first season or on the SPAD readings in the second season.

#### Response of growth–physiological attributes in salt-stressed soybean plants to the interaction between soil-applied UP and foliar-applied MgONPs

The results, as observed in Table 4, demonstrate the enhanced impact of interaction between UP rates and MgONP doses on some growth and physiological traits. Similar findings were obtained for PH, LA, and DrM% in plants fertilized with UP_3_ and sprayed with MgONP_2_ (T_32_), plants treated with UP_3_ and foliarly sprayed with MgONP_1_ (T_31_), and plants fertilized with UP_1_ combined with MgONP_2_ (T_12_) treatments produced the maximum values in PH (81.33 vs. 83.37 cm), LA (44.92 vs. 54.63 cm²), and DrM% (65.41 vs. 62.51%) in the first and second seasons, respectively. Dissimilar data were recorded regarding the highest values in SPAD readings (53.25 vs. 83.37); however, in both seasons, the plants treated with T_12_ and T_32_ produced the best results. On the hand, the minimum values in PH (44.00 cm) in the second seasons and in SPAD readings (39.46) in the first season were obtained in plants fertilized with UP_1_ combined with MgONP_1_ (T_11_). Meanwhile, plants treated with UP_2_ and MgONP_1_ (T_21_) produced the lowest %DrM (38.33 and 38.40%) in both seasons. In addition, the lowest values in LA (15.65 cm²) in the 2022 season and in SPAD readings (40.00) in the 2023 season were recorded in plants fertilized with UP_1_ only, without using MgONP (T_10_). Furthermore, applying UP_4_ with MgONP (T_42_) was the least impactful on LA in the second season. There were highly significant differences among the treatments in SPAD readings in the first season and in LA in the second season. Non-significant differences were observed in SPAD readings in the second season, in LA in the first season, as well as in PH and %DrM in both seasons.

**Table 4:** Impact of the interaction between urea-phosphate (UP) as a soil application and magnesium oxide nanoparticles (MgONPs) as a foliar nourishment on the growth-physiological attributes of soybean plants cultivated in saline-sodic soil during two consecutive seasons (2022 and 2023)

UP	MgONPs	Symbol	SPAD reading	PH	LA	DrM
				(cm)	(cm^2^)	(%)
2022 growth season						
UP_1_	MgONP_0_	T_10_	42.89±1.10de	54.33±1.43bc	15.65±1.05c	58.42±1.93ab
	MgONP_1_	T_11_	39.46±1.13e	56.00±0.99bc	20.40±1.22bc	48.83±1.06bc
	MgONP_2_	T_12_	53.25±1.22a	71.67±1.12ab	17.97±1.32bc	65.41±1.23a
UP_2_	MgONP_0_	T_20_	42.49±1.28de	63.00±1.98bc	24.85±1.10bc	40.32±1.56c
	MgONP_1_	T_21_	42.04±1.11de	57.00±1.11bc	25.58±0.97bc	38.33±1.34c
	MgONP_2_	T_22_	42.87±1.12de	50.67±1.34c	30.22±1.37b	48.85±1.84bc
UP_3_	MgONP_0_	T_30_	42.96±1.10de	63.33±1.45bc	30.93±1.10b	51.68±1.23a-c
	MgONP_1_	T_31_	46.09±1.34b-d	71.67±1.29ab	44.92±1.18a	45.28±1.11bc
	MgONP_2_	T_32_	48.09±1.36bc	81.33±1.39a	26.82±1.17bc	51.34±1.67a-c
UP_4_	MgONP_0_	T_40_	46.53±1.25b-d	49.33±1.45c	26.29±1.22bc	53.40±1.56a-c
	MgONP_1_	T_41_	49.23±1.47ab	60.33±1.01bc	23.82±1.13bc	53.16±1.38a-c
	MgONP_2_	T_42_	43.77±1.34c-e	52.67±1.41c	20.20±1.54bc	58.54±1.45ab
*p* -value						
UP (A)			0.257^ns^	0.209^ns^	0.054^*^	0.219^ns^
MgONP (B)			0.010^**^	0.224^ns^	0.188^ns^	0.032^*^
UP (A) x MgONP (B)			0.000^**^	0.057^*^	0.147^ns^	0.826^ns^
2023 growth season						
UP_1_	MgONP_0_	T_10_	44.54±1.18d	45.33±1.74b	18.23±1.65ef	59.20±1.10ab
	MgONP_1_	T_11_	48.69±1.29 cd	40.00±1.76b	21.47±1.23de	48.99±1.12bc
	MgONP_2_	T_12_	64.00±1.12bc	45.67±1.65b	19.67±1.02ef	65.21±1.23a
P_2_	MgONP_0_	T_20_	48.81±1.38 cd	50.67±1.32ab	28.17±1.92ef	39.70±1.09c
	MgONP_1_	T_21_	52.07±1.23 cd	46.67±1.11b	27.87±1.05bc	38.40±1.82c
	MgONP_2_	T_22_	64.14±1.12bc	55.67±1.10ab	30.03±1.26bc	48.03±1.10bc
UP_3_	MgONP_0_	T_30_	60.44±1.26b-d	43.67±1.23ab	33.39±1.37b	51.77±1.29a-c
	MgONP_1_	T_31_	83.37±1.92a	55.00±1.14ab	54.63±1.01a	45.25±1.05bc
	MgONP_2_	T_32_	81.60±1.61a	62.33±1.31a	31.13±1.27bc	51.30±1.05a-c
UP_4_	MgONP_0_	T_40_	63.48±1.07bc	49.33±1.29ab	25.93±1.34 cd	53.65±1.37a-c
	MgONP_1_	T_41_	70.21±1.09ab	51.00±1.90ab	22.08±1.45de	52.75±1.94a-c
	MgONP_2_	T_42_	60.55±1.37b-d	40.33±1.73b	15.64±1.87f	58.72±1.93ab
*p* -value						
UP (A)			0.041^*^	0.607^ns^	0.001^**^	0.195^ns^
MgONPs (B)			0.009^**^	0.494^ns^	0.001^**^	0.028^*^
UP (A) x MgONP (B)			0.126^ns^	0.098^*^	0.001^**^	0.817^ns^

The data are means ± SE (Standard Error) for three replicates. The mean values with different lowercase letters during each season are significant at *p* ≤ 0.05 according to Duncan’s multiple range test. SPAD reading relative chlorophyll content, PH Plant height, LA leaf area, and DrM% dry matter percentage. UP_1_, UP_2_, UP_3_, and UP_4_ represent urea-phosphate at 85.0, 107.0, 127.0 and 150.0 kg ha^−1^, respectively. MgONP_0_, MgONP_1_, and MgONP_2_ represent magnesium oxide nanoparticles at 0.00, 50.0, and 100.0 mg L^−1^, respectively

### Leaf nutrient content

#### Response of leaf nutrient content in salt-stressed soybean plants to UP soil application

**Fig. 4:**
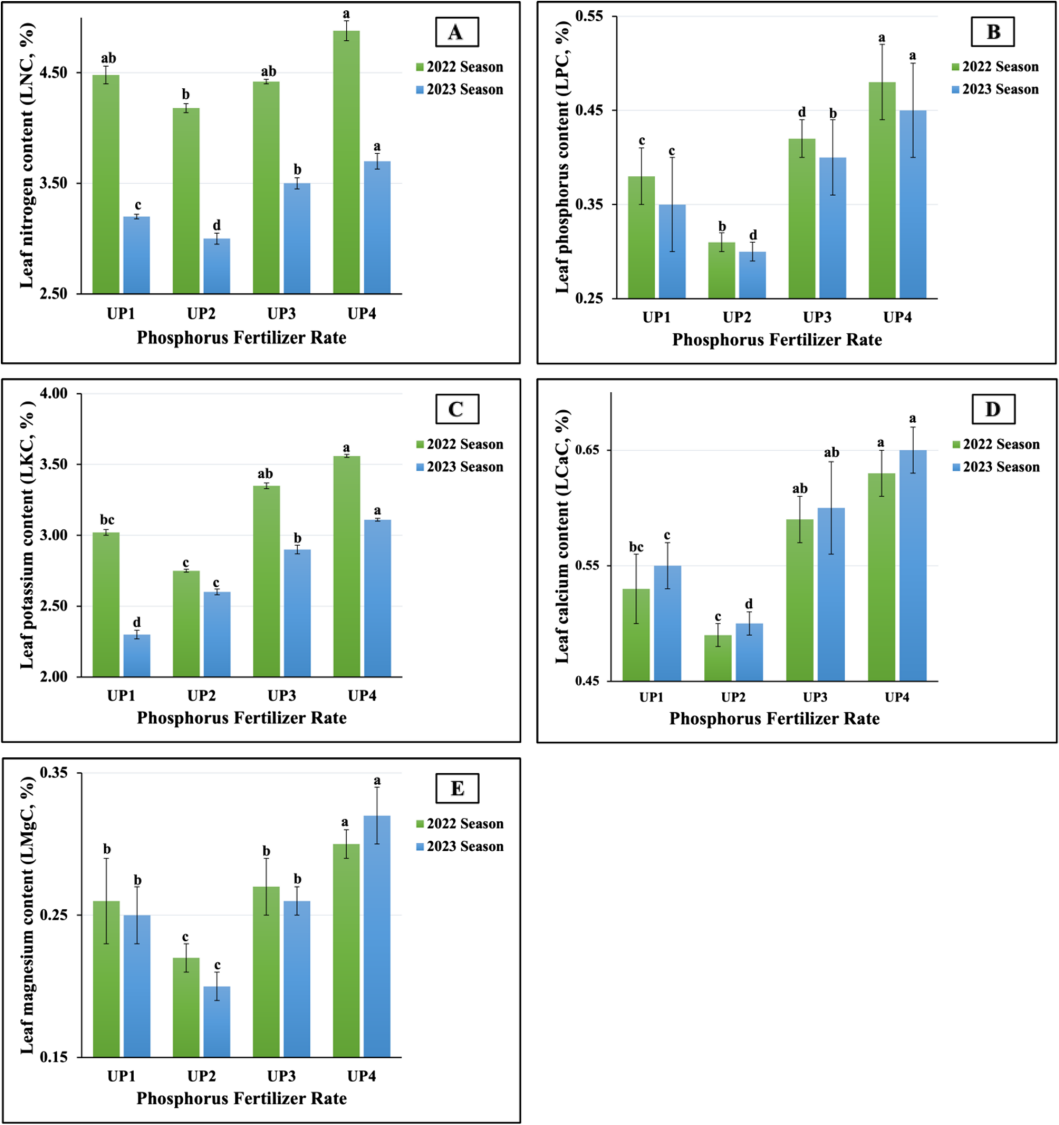
**A**-**E** The individual impact of urea-phosphate types (UPs) on leaf macronutrients content; (3A) nitrogen (LNC), (3B) phosphorus (LPC), (3C) potassium (LKC), (3D) calcium (LCaC), and (1E) magnesium (LMgC) of soybean plants cultivated in saline soil in two growing seasons 2022 and 2023 growing seasons. The data are means ± SE (Standard Error) for three replicates. Means value that have different lower-case letter in each season are significant at *p*≤0.05 according to Duncan’s multiple range test

The results, as graphically presented in Fig. 4(A-E), indicated that UP_4_ was the best application rate for soybean leaf nitrogen (LNC), phosphorus (LPC), potassium (LKC), calcium (LCaC), and magnesium (LMgC). However, this treatment produced the maximum values of 4.88 vs. 3.70% for LNC, 0.48 vs. 0.45% for LPC, 3.56 vs. 3.11% for LKC, 0.63 vs. 0.65% for LCaC, and 0.30 vs. 0.32% for LMgC in the first and second seasons, respectively. In addition, the plant leaves fertilized with UP_1_ and UP_2_ recorded the highest leaf sodium values (LNaC), recording 0.04% in both seasons, respectively. In contrast, the lowest leaf contents of N (4.18 vs. 3.00%), P (0.31 vs. 0.30%), Ca (0.49 vs. 0.50%), and Mg (0.22 vs. 0.20%) were recorded in plants fertilized with UP_1_ and UP_2_ in the 2022 and 2023 growth seasons, respectively. Moreover, UP_2_ and UP_1_ for LKC as well as UP_4_ and UP_3_ for LNaC, were the least impactful, demonstrating 2.75 vs. 2.30% and 0.03 vs. 0.02% for both elements in both seasons, respectively. For all the UP rates tested, highly significant differences were obtained for the above-mentioned nutrients, except for LNaC, which had a non-significant effect in both seasons.

**Fig. 5:**
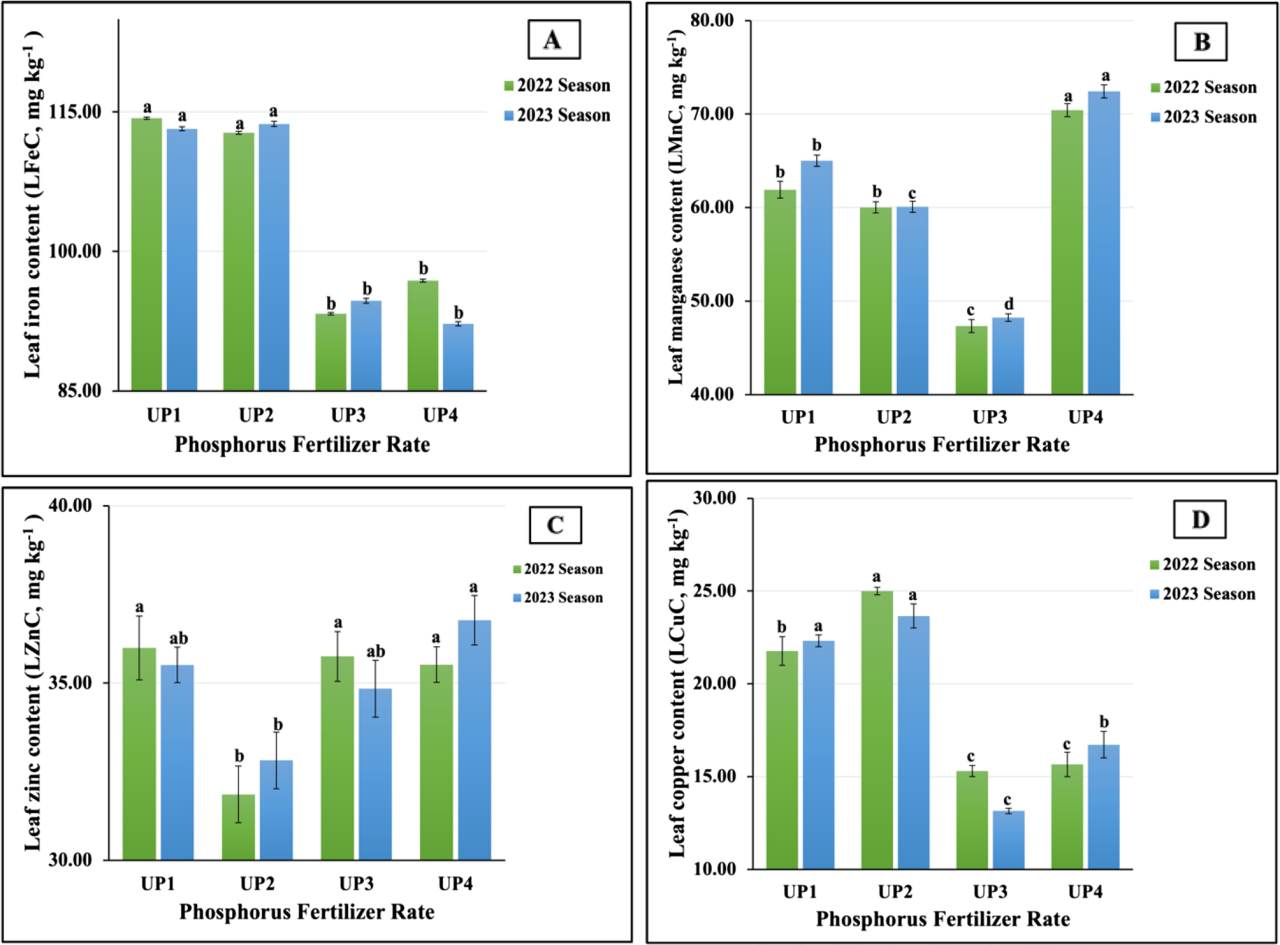
**A**-**D** The individual impact of urea-phosphate rates (UPs) on leaf micronutrients content; (4A) iron (LFeC), (4B) manganese (LMnC), (4 C) zinc (LZnC), and (4D) copper (LCuC) of soybean plants cultivated in saline soil in the 2022 and 2023 growing seasons. The data are means ± SE (Standard Error) for three replicates. Means value that have different lower-case letter in each season are significant at *p* ≤ 0.05 according to Duncan’s multiple range test

As seen in Fig. 5(A-D), the results related to the impact of UP fertilizer rates on the content of leaf micronutrients, such as iron (LFeC), manganese (LMnC), zinc (LZnC), and copper (LCuC), demonstrated that the plants fertilized with UP_4_ produced the maximum content of Mn (70.42 vs. 72.43 mg kg^−1^) in both seasons and of LZnC (36.77 mg kg^−1^) in the second season. Meanwhile, the plants treated with UP_2_ produced the highest LCuC (25.00 vs. 23.65 mg kg^−1^) in both growing seasons and the highest LFeC in the second season (113.72 mg kg^1^). Furthermore, UP was the most influential on LFeC (114.33 mg kg^−1^) and LZnC (35.99 mg kg^−1^) in the first season. On the contrary, UP_3_ were the least impactful, demonstrating the minimum values in LMnC (47.33 vs. 48.22 mg kg^−1^ and LCuC (15.30 vs. 13.15 mg kg^−1^) in both growing seasons, respectively, and the minimum value in LFeC (93.30 mg kg^−1^) in the first season. The lowest LZnC (31.86 vs. 32.82 mg kg^−1^ in 2022 and 2023, respectively) was obtained in plants treated with UP_2_ in both growth seasons. Statistically, highly significant differences were found for LFeC, LMnC, and LCuC; moreover, non-significant effects were found for LZnC in both growth seasons.

#### Response of leaf nutrient content in salt-stressed soybean plants to MgONPs foliar application

**Fig. 6:**
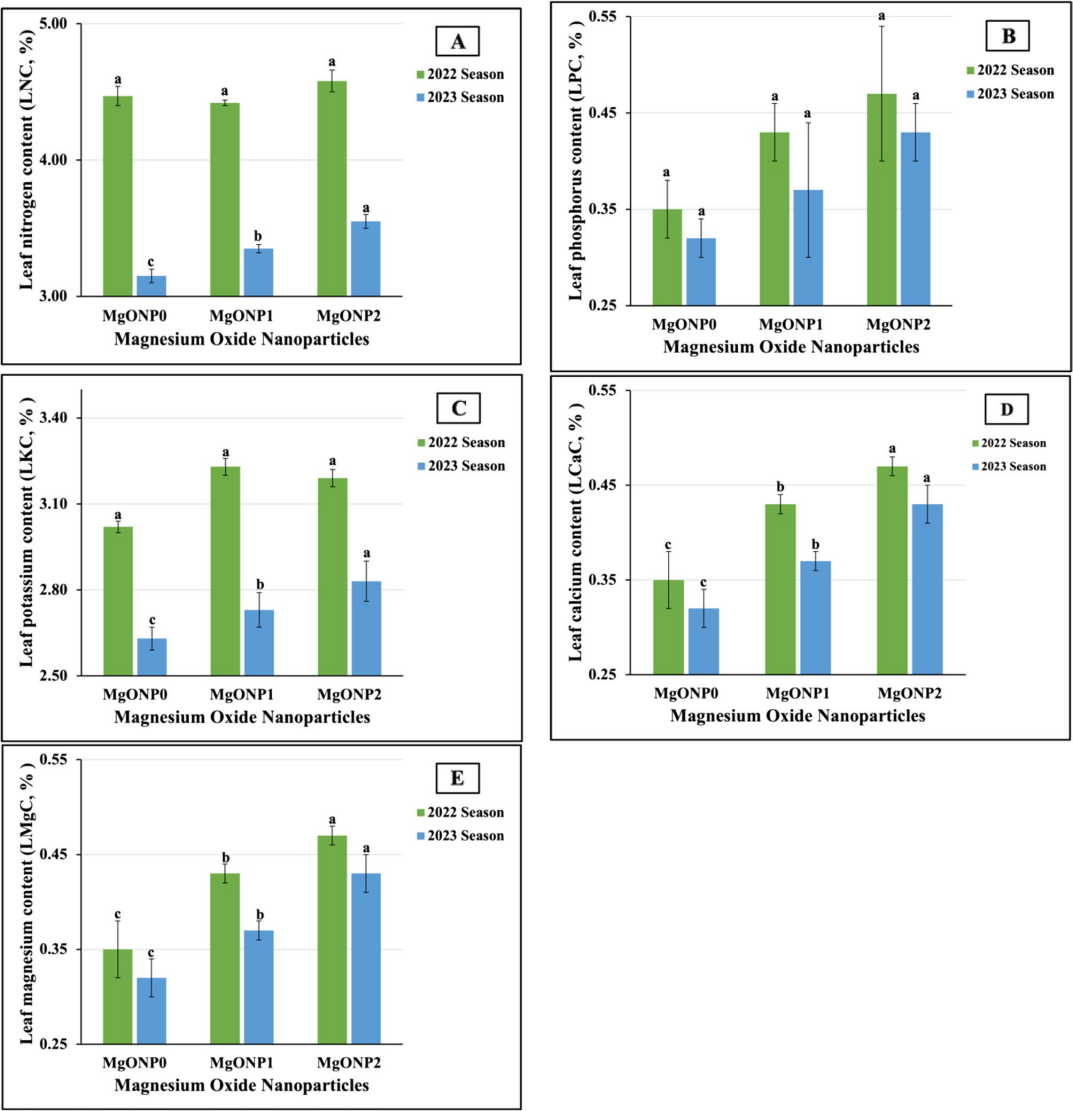
**A**-**E** The individual impact of magnesium oxide nanoparticle doses (MgONPs) on leaf macronutrients content; (3A) nitrogen (LNC), (3B) phosphorus (LPC), (3C) potassium (LKC), (3D) calcium (LCaC), and (1E) magnesium (LMgC) of soybean plants cultivated in saline soil in two growing seasons 2022 and 2023 growing seasons. The data are means ± SE (Standard Error) for three replicates. Means value that have different lower-case letter in each season are significant at *p*≤0.05 according to Duncan’s multiple range test

The impact of the application of MgONP doses on the leaf contents of the aforementioned macronutrients in the 2022 and 2023 seasons are graphically presented in Fig. 6(A-E). Similar findings were obtained for LPC, LCaC, and LMgC. The MgONP doses, ranked in descending order in terms of MgONP_2_ > MgONP_1_ > MgONP_0_, were 0.47 > 0.41 > 0.35 and 0.43 > 0.37 > 0.32 for LPC, 0.62 > 0.56 > 0.51 and 0.63 > 0.58 > 0.63 for LCaC, and 0.31 > 0.27 > 0.22 and 0.30 > 0.26 > 0.20 for LMgC in each growth season, respectively. With regard to LNaC, the doses of MgONPs were arranged in the following order (for MgONP_0_ > MgONP_1_ > MgONP_2_): 0.04 > 0.03 > 0.02 and 0.04 > 0.04 > 0.03 in the 2022 and 2023 growing seasons, respectively. Dissimilar results were achieved in both growth seasons for LNC and LKC. However, the results for treatment with MgONPs were ranked as, in descending order, MgONP_2_ (4.58%) > MgONP_0_ (4.47%) > MgONP_1_ (4.42%) and MgONP_2_ (3.55%) > MgONP_1_ (3.35%) > MgONP_0_ (3.15%) for LNC and as MgONP_1_ (3.23%) > MgONP_2_ (3.19%) > MgONP_0_ (3.09%) and MgONP_2_, (2.83%) > MgONP_1_ (273%) > MgONP_0_ (2.63%) for LKC in the 2022 and 2023 seasons, respectively.

**Fig. 7:**
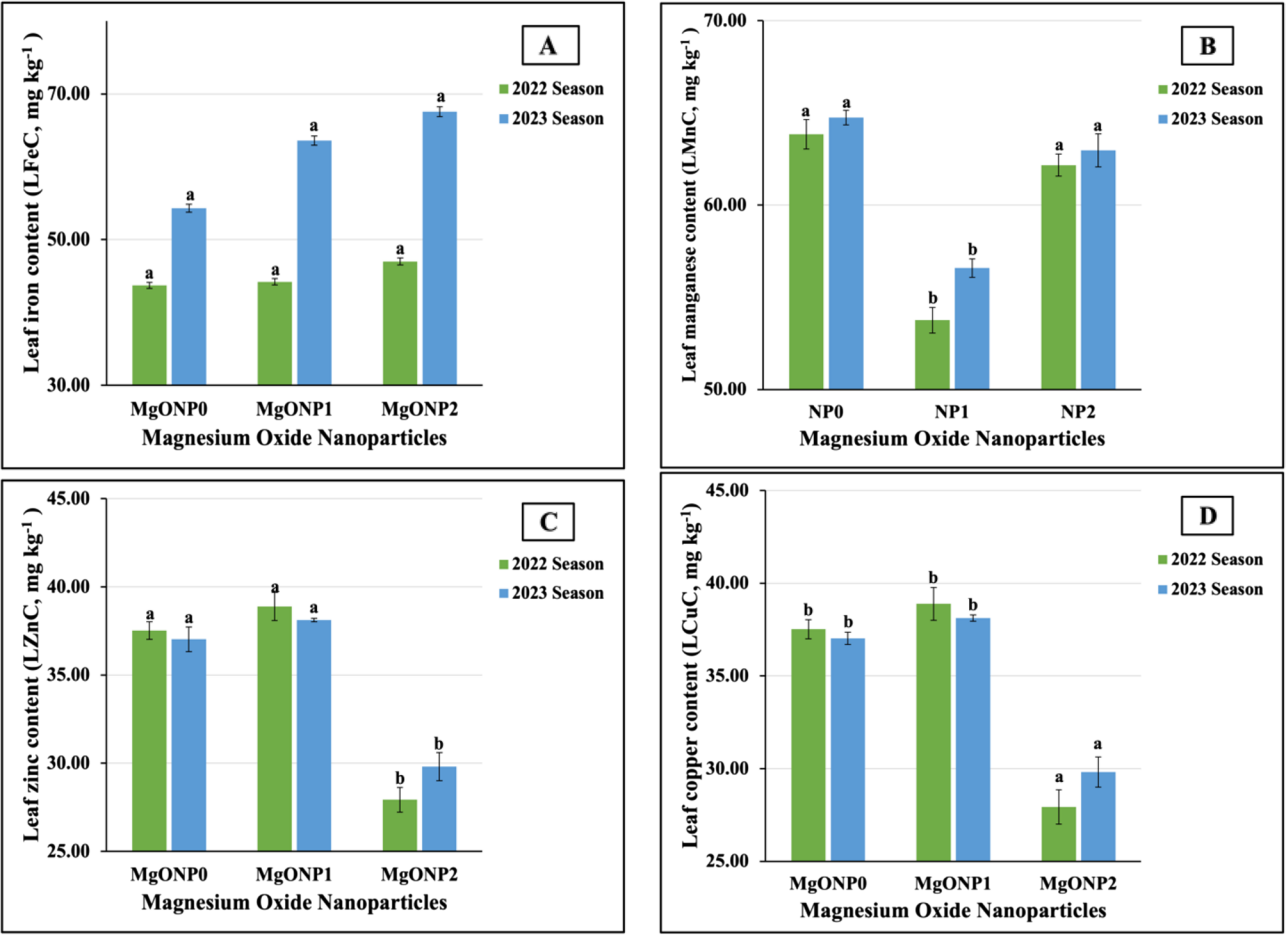
**A**-**D** The individual impact of magnesium oxide nanoparticle doses (MgONPs) on leaf micronutrients content; (6A) iron (LFeC), (6B) manganese (LMnC), (6C) zinc (LZnC), and (6D) copper (LCuC) of soybean plants cultivated in saline soil in the 2022 and 2023 growing seasons. The data are means ± SE (Standard Error) for three replicates. Means value that have different lower-case letter in each season are significant at *p*≤0.05 according to Duncan’s multiple range test

The results obtained from the ANOVA showed that MgONPs had highly significant influences on the leaf content of P, Ca, and Mg in both seasons, of LNC and LKC in the 2023 season, and of LNC in the 2022 season. Non-significant differences were found for LNC and LKC in the first season. The results depicted in Fig. 7(A-D) document the effect of treatment with different MgONPs on the micronutrient contents of soybean leaves during the 2022 and 2023 seasons.

The statistical analysis revealed that MgONPs did not have a significant impact on LFeC and had highly significant influences on LMnC, LZnC, and LCuC in both growth seasons. The obtained results demonstrated that the highest LMnC values (63.84 vs. 64.75 mg kg^−1^) were produced in untreated plants. Meanwhile, the plants treated with MgONP_2_ and MgONP_1_ produced the maximum LFeC in two growing seasons. Regarding the highest values of LZnC and LCuC, our results noted that MgONP_1_ for LZnC and MgONP_2_ for LCuC were the most impactful, producing levels of 38.89 vs. 38.12 mg kg^1^ and 23.08 vs. 22.10 mg kg^−1^ in the 2022 and 2023 seasons, respectively. On the contrary, the lowest LFeC (100.64 vs. 103.04 mg kg^−1^) and LCuC (16.91 vs. 17.26 mg k*g*^−1^) were obtained in untreated plants. Furthermore, the least influence of fertilizer on LMnC and LZnC levels was found in plants fertilized with MgONP_1_ and MgONP_2_, respectively; the minimum values in both growing seasons were recorded in these plants.

#### Response of leaf nutrient content in salt-stressed soybean plants to the interaction between soil-applied UP and foliar-applied MgONPs

Despite the improvements obtained due to the interactive impact between UP and MgONP, the results obtained from the statistical analysis indicated that there were no significant effects on all the macronutrients studied. The finding obtained from our field study highlighted the pivotal influence of using maximum rates of both UP and MgONP_5_, as listed in Table 5. More clearly, the maximum contents of N (5.52 vs. 3.90% in the 2022 and 2023 seasons, respectively), P (0.54 vs. 6.50% in the 2022 and 2023 seasons, respectively), Ca (0.67 vs. 0.70% in the 2022 and 2023 seasons, respectively), and Mg (0.36 vs. 0.34% in the 2022 and 2023 seasons, respectively) were recorded in soybean plants fertilized with UP_4_ and foliarly sprayed with MgONP_2_. Similarly, plants treated T_20_ produced the highest values in Na (0.05 vs. 0.04% in 2022 vs. 2023) in both seasons. For LKC, dissimilar results were produced among both growth seasons, as the highest values of K were found in the leaves of plants treated with T_41_ (3.64%) in the first season and in those treated with T_42_ (3.20%) in the second season. As for the lowest values obtained, the results were completely different. However, the T_20_ treatment was the least influential for LCaC (0.44 vs. 0.45%), LMgC (0.20 vs. 0.19%), and LPC (0.29 vs. 0.25%) in both growing seasons and for LNC (2.80%) and LKC (2.20%) in the second season. Furthermore, the application of T_21_ produced the lowest values in LNC (3.70%) and LKC (2.66%) in the first season, while plants treated with T_42_ produced the minimum LNaC values (0.01 vs. 0.02% in the 2022 and 2023 growth seasons, respectively). It is clear from Table 6 that the interaction between UP and MgONP significantly affected the leaf micronutrient content in soybean plants. The obtained results indicated that the application of T_20_ and T_10_ treatments was the most influential on LFeC (140.01 vs. 144.71 mg kg^−1^) and LMnC (93.43 vs. 96.87 mg kg^−1^) in the 2022 and 2023 seasons, respectively. Dissimilar results were found for LZnC and LCuC during both growth seasons. However, the maximum values in LZnC (52.32 vs. 51.75 mg kg^−1^) were produced in plants treated with T_31_ and T_11_. Likewise, the plants treated with T_12_ and T_11_ demonstrated the highest LCuC values (30.00 vs. 29.42 mg kg^−1^) in both seasons. In spite of the clear variation in the best values obtained, the values associated with the lowest were similar to each other. However, the plants treated with T_40_, T_32_, and T_31_ demonstrated the lowest values in Fe (53.24 vs. 51.50 mg kg^−1^), Mn (22.80 vs. 21.68 mg kg^−1^), and Cu (19.20 vs. 8.20 mg kg^−1^) in the first and second seasons. The application of T_30_, and T_12_ treatments was the least impactful on LZnC levels of 22.27 and 21.37 mg kg^−1^ were recorded in the 2022 and 2023 seasons, respectively.

**Table 5:** Impact of the interaction between urea-phosphate (UP) as a soil application and magnesium oxide nanoparticles (MgONPs) as a foliar nourishment on the leaf macronutrient contents of soybean plants cultivated in saline-sodic soil during two consecutive seasons (2022 and 2023)

UP	MgONP	Symbol	LNC	LPC	LKC	LCaC	LMgC
			(%)				
2022 growth season							
UP_1_	MgONP_0_	T_10_	4.24±0.02bc	0.33±0.02e	2.93±0.02b-d	0.49±0.01e	0.21±0.02e
	MgONP_1_	T_11_	4.91±0.01ab	0.38±0.01d	3.11±0.03a-d	0.54±0.02 cd	0.25±0.02 cd
	MgONP_2_	T_12_	4.23±0.01bc	0.43±0.02c	3.04±0.03a-d	0.59±0.02bc	0.30±0.01b
UP_2_	MgONP_0_	T_20_	4.63±0.02ab	0.29±0.01f	2.88±0.03 cd	0.44±0.03e	0.19±0.01e
	MgONP_1_	T_21_	3.72±0.03c	0.31±0.03e	2.66±0.02d	0.51±0.04de	0.23±0.01de
	MgONP_2_	T_22_	4.16±0.03bc	0.42±0.03d	2.70±0.02d	0.56±0.02 cd	0.25±0.02 cd
UP_3_	MgONP_0_	T_30_	4.37±0.02bc	0.39±0.02d	3.08±0.02a-d	0.54±0.02 cd	0.23±0.02de
	MgONP_1_	T_31_	4.61±0.02bc	0.45±0.01c	3.52±0.03ab	0.59±0.01bc	0.28±0.02bc
	MgONP_2_	T_32_	4.38±0.01bc	0.49±0.01b	3.46±0.01a-c	0.66±0.02ab	0.31±0.01b
UP_4_	MgONP_0_	T_40_	4.61±0.02bc	0.43±0.01c	3.48±0.02a-c	0.59±0.02bc	0.23±0.03de
	MgONP_1_	T_41_	4.49±0.01bc	0.51±0.03b	3.64±0.02a	0.64±0.03ab	0.31±0.02b
	MgONP_2_	T_42_	5.52±0.01a	0.54a±0.02a	3.56±0.03a	0.67±0.02a	0.36±0.02a
*p*-value							
UP (A)			0.051^*^	0.000^**^	0.012^**^	0.011^**^	0.000^**^
MgONP (B)			0.729^ns^	0.000^**^	0.554^ns^	0.000^**^	0.000^**^
UP (A) x MgONP (B)			0.036^*^	0.124^ns^	0.706^ns^	0.951^ns^	0.244^ns^
2023 growth season							
UP_1_	MgONP_0_	T_10_	4.27±0.03bc	0.30±0.02e	2.50±0.03gh	0.51±0.01de	0.20±0.02de
	MgONP_1_	T_11_	4.19±0.02ab	0.35±0.01d	2.60±0.02 fg	0.55±0.02 cd	0.25 ±0.01b-d
	MgONP_2_	T_12_	4.25±0.02bc	0.40±0.03c	2.70±0.01ef	0.66±0.02c	0.30±0.02ab
UP_2_	MgONP_0_	T_20_	4.66±0.03ab	0.25±0.02f	2.20±0.02j	0.45±0.03e	0.18±0.01e
	MgONP_1_	T_21_	3.70±0.01c	0.30±0.01e	2.30±0.01ij	0.50±0.03de	0.22 ±0.01c-d
	MgONP_2_	T_22_	4.19±0.01bc	0.35±0.02d	2.40±0.01hi	0.55±0.01 cd	0.25 ±0.02b-d
UP_3_	MgONP_0_	T_30_	4.34±0.02bc	0.35±0.02d	2.80±0.02de	0.55±0.02 cd	0.22±0.01c-e
	MgONP_1_	T_31_	4.58±0.02bc	0.40±0.02c	2.90±0.02 cd	0.60±0.02bc	0.27±0.02bc
	MgONP_2_	T_32_	4.35±0.03bc	0.45±0.01b	3.00±0.02bc	0.65±0.01ab	0.30±0.02ab
UP_4_	MgONP_0_	T_40_	4.62±0.02bc	0.40±0.01c	3.03±0.02bc	0.60±0.02bc	0.25±0.03b-d
	MgONP_1_	T_41_	4.50±0.01bc	0.45±0.03b	3.10±0.02ab	0.65±0.01ab	0.30±0.02ab
	MgONP_2_	T_42_	5.59±0.03a	0.50±0.02a	3.20±0.03a	0.70±0.02a	0.35±0.02a
*p*-value							
UP (A)			0.000^**^	0.000^**^	0.000^**^	0.008^**^	0.003^**^
MgONP (B)			0.000^**^	0.000^**^	0.000^**^	0.000^**^	0.000^**^
UP (A) x MgONP (B)			0.922^ns^	0.999^ns^	0.919^ns^	0.928^ns^	0.950^ns^

The data are means ± SE (Standard Error) for three replicates. The mean values with different lowercase letters during each season are significant at *p* ≤ 0.05 according to Duncan’s multiple range test. LNC, LPC, LKC, LCaC, and LMgC indicate the leaf nitrogen, phosphorus, potassium, calcium, and magnesium contents, respectively. UP_1_, UP_2_, UP_3_, and UP_4_ represent urea-phosphate at 85.0, 107.0, 127.0 and 150.0 kg ha^−1^, respectively. MgONP_0_, MgONP_1_, and MgONP_2_ represent magnesium oxide nanoparticles at 0.00, 50.0, and 100.0 mg L^−1^, respectively

**Table 6:** Impact of the interaction between urea-phosphate (UP) as a soil application and magnesium oxide nanoparticles (MgONPs) as a foliar nourishment on the leaf micronutrient contents of soybean plants cultivated in saline-sodic soil during two consecutive seasons (2022 and 2023)

UP	MgONP	Symbol	LFeC	LMnC	LZnC	LCuC
			(mg kg^−1^)			
2022 growth season						
UP_1_	MgONP_0_	T_10_	125.42±0.10bc	93.43±0.70a	34.09±0.50c	10.66±0.50f
	MgONP_1_	T_11_	114.7±0.20 cd	27.77±0.60 h	51.57±0.40a	25.64 ±0.30a-c
	MgONP_2_	T_12_	102.60±0.10e	64.55±0.54e	22.30±0.30e	30.00a±0.20
UP_2_	MgONP_0_	T_20_	140.01±0.10a	44.36±0.91f	44.13±0.70b	25.05±0.20a-c
	MgONP_1_	T_21_	130.41±0.10ab	62.84±0.80e	28.63±0.60d	27.08±0.20ab
	MgONP_2_	T_22_	67.86±0.10 g	72.85±81d	22.83±0.50e	22.87±0.30b-d
UP_3_	MgONP_0_	T_30_	83.88±0.20f	85.05±0.60c	22.27±0.30e	18.70±0.40de
	MgONP_1_	T_31_	67.95±0.20 g	34.13±0.70 g	52.32±0.50a	10.20±0.80f
	MgONP_2_	T_32_	126.08±0.20bc	22.80±0.60i	32.65±0.50 cd	18.00±0.20de
UP_4_	MgONP_0_	T_40_	53.24±0.10 h	32.51±0.57 g	49.58±0.50a	14.23±0.30ef
	MgONP_1_	T_41_	108.52±0.20de	90.29±0.78ab	23.05±0.50e	11.28±0.40f
	MgONP_2_	T_42_	128.80±0.10ab	88.47±0.68bc	33.93±0.40c	21.46±0.20 cd
*p*-value						
UP (A)			0.000^**^	0.000^**^	0.092^ns^	0.000^**^
MgONP (B)			0.056^*^	0.000^**^	0.000^**^	0.000^**^
UP (A) x MgONP (B)			0.000^**^	0.000^**^	0.000^**^	0.000^**^
2023 growth season						
UP_1_	MgONP_0_	T_10_	132.10±0.20b	96.87±0.50a	33.42±0.20de	10.33±0.50 fg
	MgONP_1_	T_11_	108.36±0.20c	30.13±0.40 h	51.75±0.30a	29.42±0.20a
	MgONP_2_	T_12_	99.12±0.20c	68.02±0.50e	21.37±0.40f	27.23±0.30ab
UP_2_	MgONP_0_	T_20_	144.71±0.20a	43.08±0.50f	43.03±0.50c	26.42±0.40a-c
	MgONP_1_	T_21_	130.45±0.20b	63.72±0.40e	30.69±0.30e	23.12±0.50bc
	MgONP_2_	T_22_	66.00±0.10e	73.47±0.40d	24.73±0.40f	21.41±0.50 cd
UP_3_	MgONP_0_	T_30_	83.84±0.10d	83.51±0.50c	22.04±0.50f	15.43±0.50ef
	MgONP_1_	T_31_	73.05±0.20de	39.29±0.60 fg	45.39±0.60bc	10.28±0.50 g
	MgONP_2_	T_32_	127.19±0.20b	21.86±0.70i	37.11±0.60d	15.74±0.60ef
UP_4_	MgONP_0_	T_40_	51.50±0.10f	35.56±0.40 g	49.64±0.70ab	16.88±0.60de
	MgONP_1_	T_41_	107.55±0.10c	93.21±0.50ab	24.64±0.60f	10.27±0.70 g
	MgONP_2_	T_42_	120.62±0.10b	88.53±0.20b	36.02±0.40de	24.01±0.40a-c
*p*-value						
UP (A)			0.000^**^	0.000^**^	0.162^ns^	0.000^**^
MgONP (B)			0.786^ns^	0.000^**^	0.000^**^	0.002^**^
UP (A) x MgONP (B)			0.000^**^	0.000^**^	0.000^**^	0.000^**^

The data are means ± SE (Standard Error) for three replicates. The mean values with different lowercase letters during each season are significant at *p* ≤ 0.05 according to Duncan’s multiple range test. LFeC, LMnC, LZnC, and LCuC indicate the leaf iron, manganese, zinc, and copper contents, respectively. UP_1_, UP_2_, UP_3_, and UP_4_ represent urea-phosphate at 85.0, 107.0, 127.0 and 150.0 kg ha^−1^, respectively. MgONP_0_, MgONP_1_, and MgONP_2_ represent magnesium oxide nanoparticles at 0.00, 50.0, and 100.0 mg L^−1^, respectively

### Seeds’ mineral compositions

#### Response of mineral seed composition in salt-stressed soybean plants to UP soil application

**Fig. 8:**
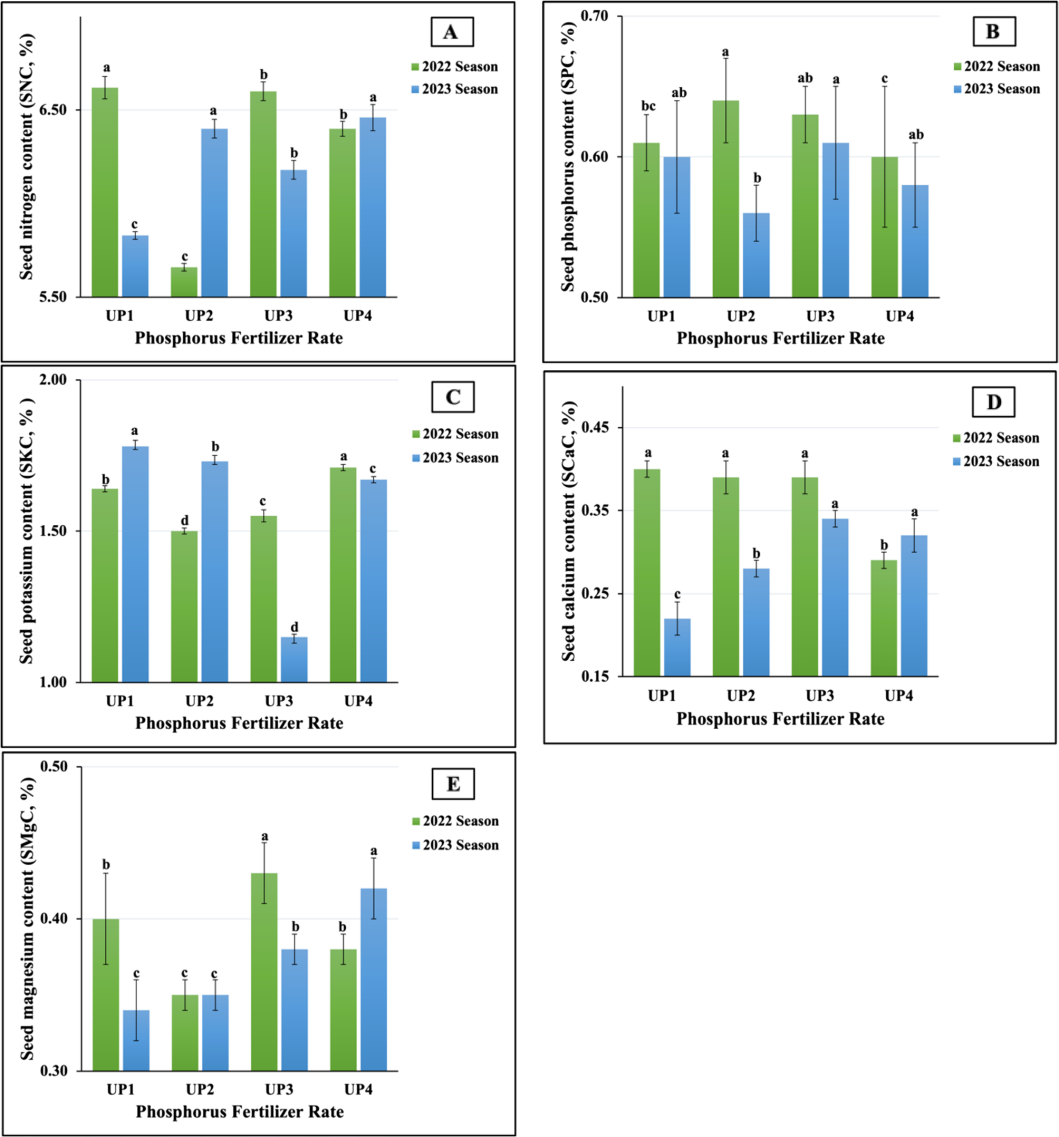
**A**-**E** The individual impact of urea-phosphate types (UPs) on seed macronutrients content; (8A) nitrogen (SNC), (8B) phosphorus (SPC), (8C) potassium (SKC), (8D) calcium (SCaC), and (8E) magnesium (SMgC) of soybean plants cultivated in saline soil in two growing seasons 2022 and 2023 growing seasons. The data are means ± SE (Standard Error) for three replicates. Means value that have different lower-case letter in each season are significant at *p*≤0.05 according to Duncan’s multiple range test

Figure 8(A-E) present the influences of the application of the different rates of UP as a soil fertilizer on the macronutrient contents of the seeds in the 2022 and 2023 seasons. According to the results, there was no noticeable benefit from adding any particular treatment over the others. However, the plants fertilized at UP_1_ produced the maximum seed nitrogen (SNC) and calcium (SCaC) contents, recording 6.62% and 0.40%, respectively, in the 2022 season, as well as a seed potassium content (SKC) of 1.78% in the 2023 season. In addition, UP₃ was the most impactful rate for the seed magnesium content (SMgC), which was recorded as 0.43% in the first season, and for the seed phosphorus (SPC) (0.61%) and SCaC (0.34%) in the second season. Meanwhile, UP_4_ was the superior rate, producing the maximum SKC value (1.71%) in the first season, as well as the highest SNC (6.46%) and SMgC (0.42%) values in the second season.

**Fig. 9:**
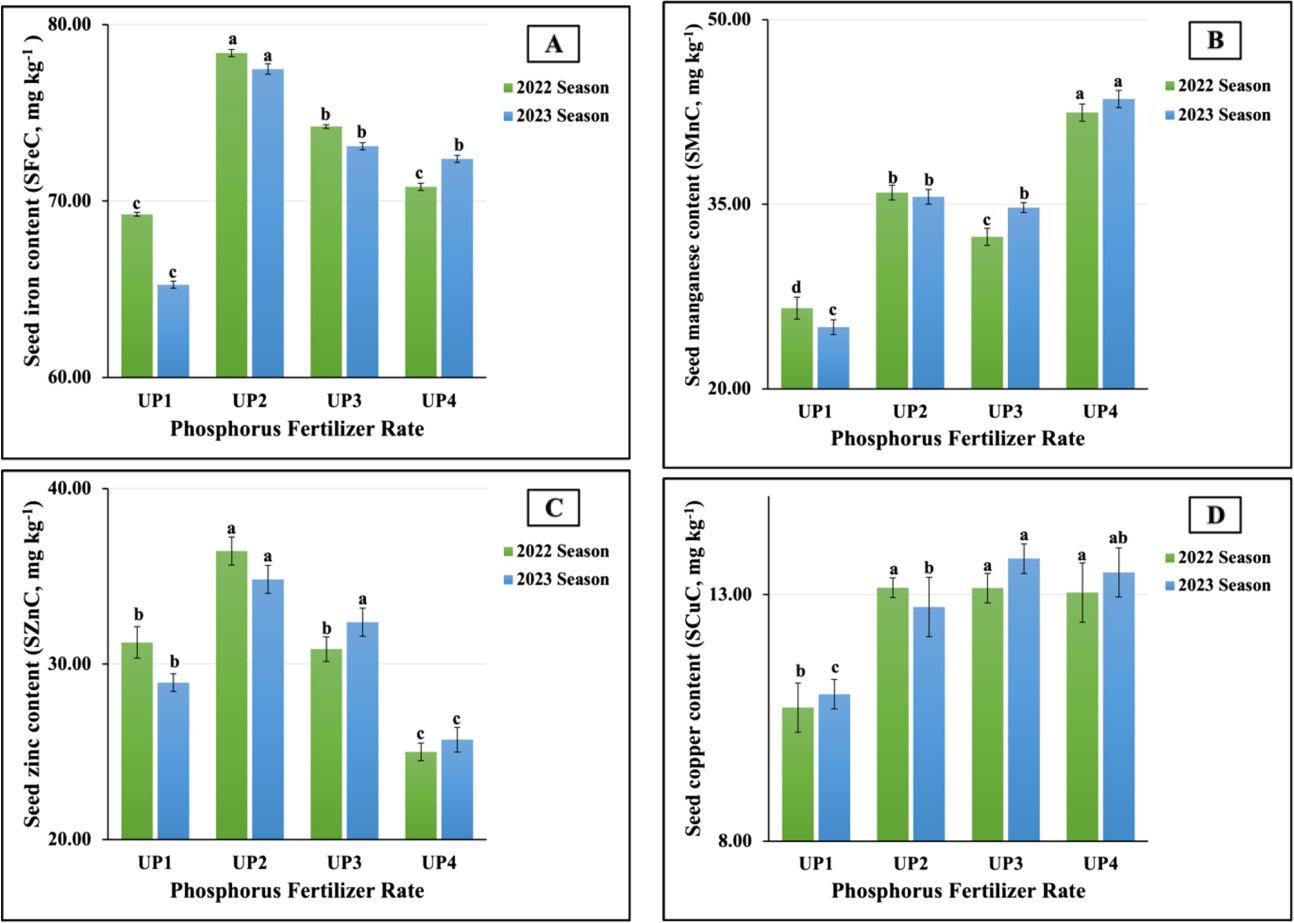
**A**-**D** The individual impact of urea-phosphate rates (UPs) on seed micronutrients content; (9A) iron (SFeC), (9B) manganese (SMnC), (9C) zinc (SZnC), and (9D) copper (SCuC) of soybean plants cultivated in saline soil in the 2022 and 2023 growing seasons. The data are means ± SE (Standard Error) for three replicates. Means value that have different lower-case letter in each season are significant at *p*≤0.05 according to Duncan’s multiple range test

In contrast, UP₂ was the least influential for the SMgC (0.35 vs. 0.33%) contents in both growth seasons, as well as for the SNC (5.66%) and SKC (1.50%) in the first season and the SPC (0.56%) in the second season. The lowest SNC (5.83%) and SCaC (0.22%) values were obtained in the plants treated at UP_1_ in the second season. Furthermore, the application of UP_4_ produced the minimum SPC (0.60%) and SCaC (0.29%) values in the first season. The results obtained from the ANOVA indicated that all the treatments had highly significant influences on the SNCs, SKCs, SCaCs, and SMgCs; in addition, significant effects on the SPCs were observed in both seasons. The results presented in Fig. 9(A-D) reveal the beneficial effect that UP₂ exerted on the micronutrient contents of the seeds. The highest iron (SFeC) and zinc (SZnC) contents were recorded in both growing seasons (78.39 vs. 77.48 mg kg⁻¹ for the SFeC and 36.44 vs. 34.82 mg kg⁻¹ for the SZnC in 2022 and 2023, respectively). Moreover, the maximum seed manganese contents (SMnCs) were recorded in the plants fertilized at UP_4_ (42.45 vs. 43.55 mg kg⁻¹ in 2022 and 2023, respectively). Dissimilar findings were obtained for the seed copper contents (SCuCs); however, the highest values were produced as a result of applying UP_2_ and UP_3_ in both seasons, 2022 and 2023, respectively. In contrast, the UP_1_ application was the least influential; the minimum SFeC (69.25 vs. 65.26 mg kg⁻¹), SMnC (26.56 vs. 25.03 mg kg⁻¹), and SCuC (10.71 vs. 10.98 mg kg⁻¹) values were recorded in the plants treated at UP_1_ in both growth seasons, respectively. Meanwhile, the lowest SZnC values were achieved in the plants treated at UP_4_ (25.00 vs. 25.69 mg kg⁻¹ in 2022 and 2023, respectively). Statistically, all the treatments had significant impacts (at *p* ≤ 0.01) for all the aforementioned micronutrients in the first and second seasons.

#### Response of mineral seed composition in salt-stressed soybean plants to MgONPs foliar application

**Fig. 10:**
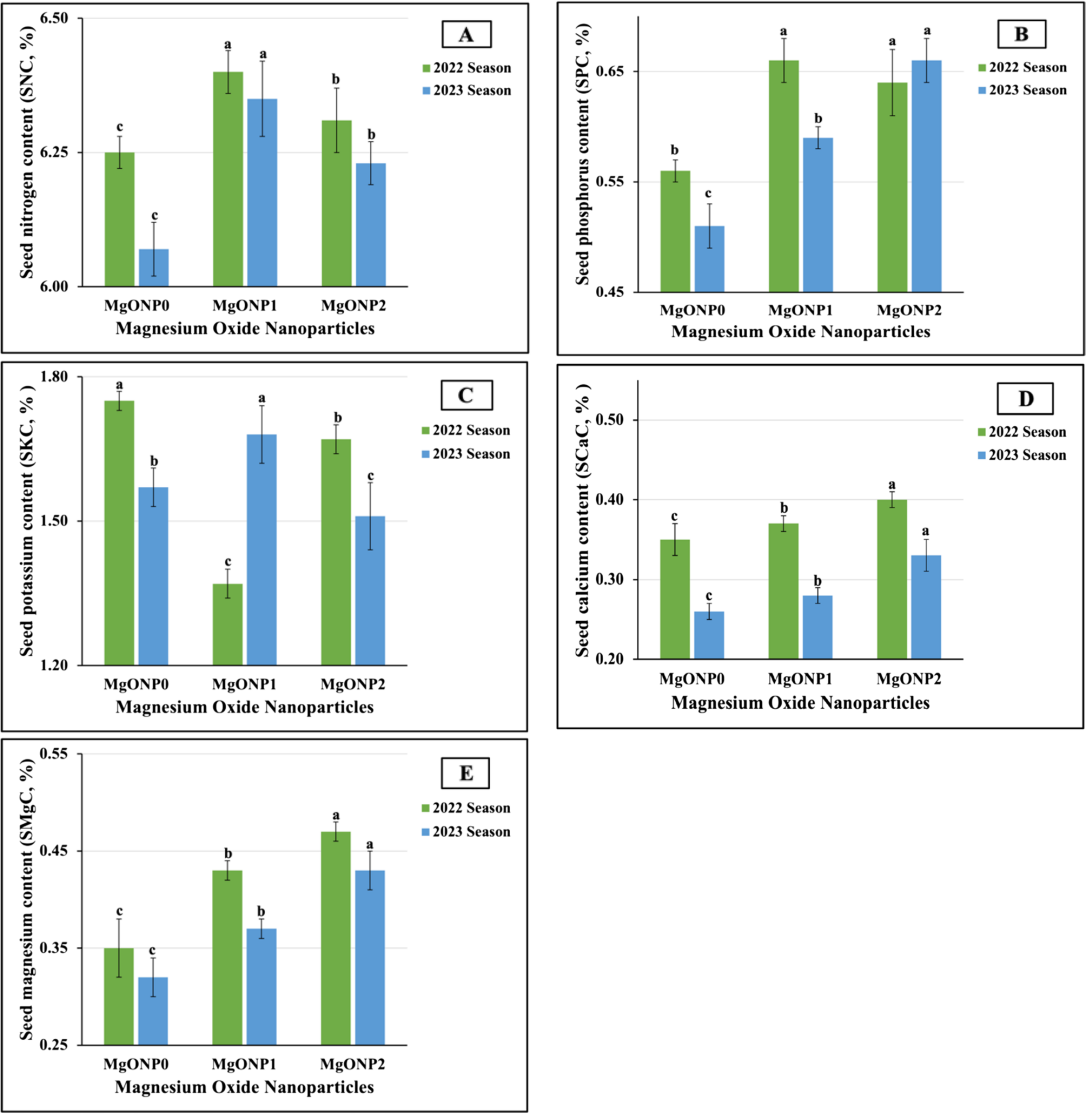
**A**-**E** The individual impact of magnesium oxide nanoparticle doses (MgONPs) on seed macronutrients content; (10A) nitrogen (SNC), (10B) phosphorus (SPC), (10C) potassium (SKC), (10D) calcium (SCaC), and (10E) magnesium (SMgC) of soybean plants cultivated in saline soil in two growing seasons 2022 and 2023 growing seasons. The data are means ± SE (Standard Error) for three replicates. Means value that have different lower-case letter in each season are significant at *p*≤0.05 according to Duncan’s multiple range test

The results obtained from the statistical analysis indicated that all MgONPs treatments had significant impacts (at *p* ≤ 0.01) on all studied macronutrient content levels in both growth seasons. As graphically presented in Fig. 10(A-E), the greatest improvements in the macronutrient content of leaves were closely associated with the application of MgONP_1_ and MgONP_2_ treatments, whereas the maximum values in SNC (6.40 vs. 6.35%) in both seasons, as well as in SPC (0.66%) in the first season and in SKC (1.68%) and SMgC (0.38%) in the second season were achieved in soybean plants that were foliar applied with MgONP_1_. Furthermore, the highest values in SCaC (0.40 vs. 0.33%) in both growing seasons and the highest value in SMgC (0.42%) in the 2022 season and in SPC (0.66%) in the 2023 season were achieved in plants treated with MgONP₂. The highest SKC value (1.75) in the first season was obtained in untreated plants. Conversely, the lowest values in SNC (6.25 vs. 6.07%), SPC (0.56 vs. 051%), SCaC (0.33 vs. 0.26), and SMgC (0.36 vs. 6.34%) were obtained in untreated plants (MgONP_0_). Dissimilar findings were produced regarding SKC, as the minimum values were recorded as a result of MgONP_1_ in the first season and as a result of MgONP_2_ in the second season.

**Fig. 11:**
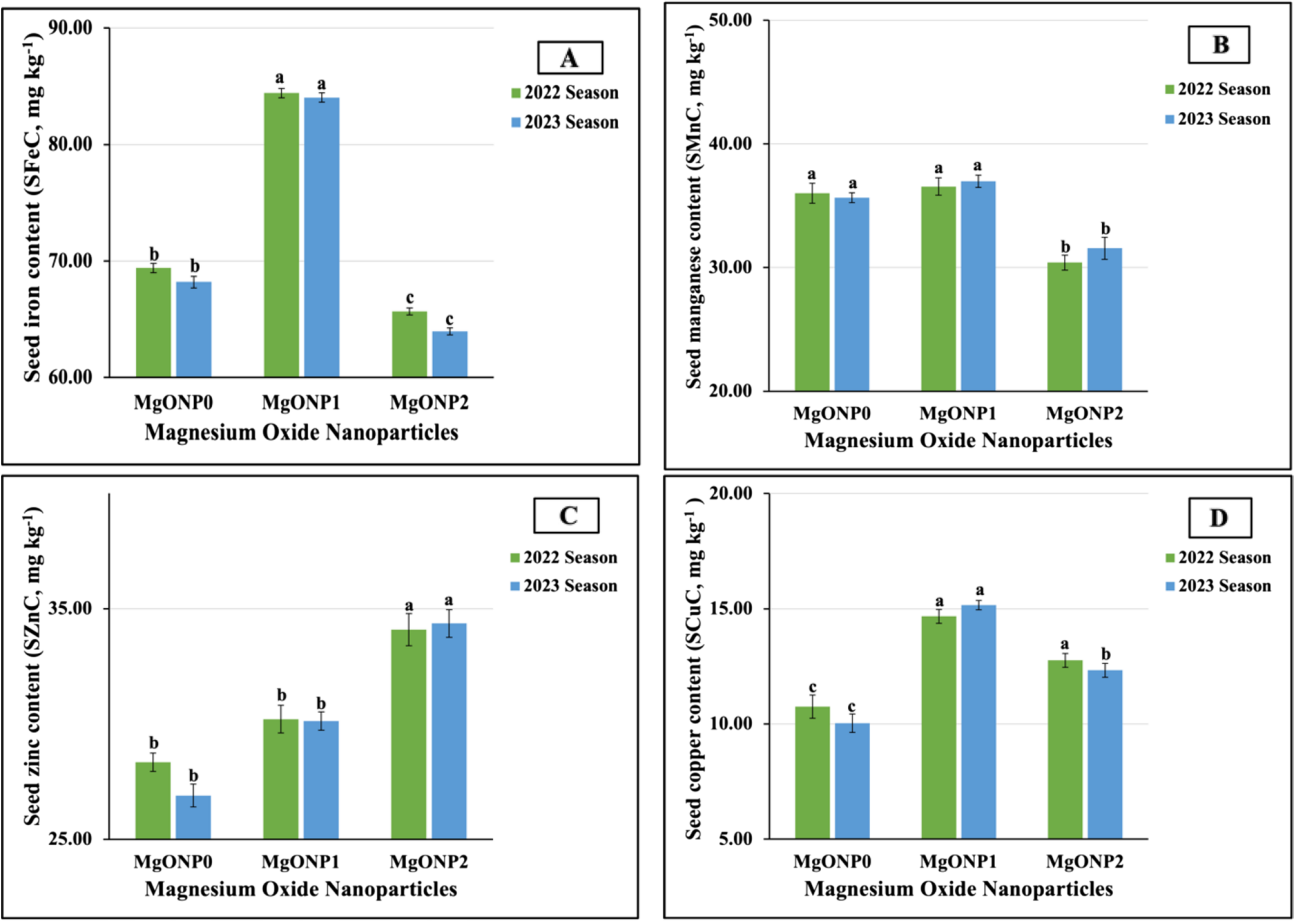
**A**-**D **The individual impact of magnesium oxide nanoparticle doses (MgONPs) on seed micronutrients content; (11A) iron (SFeC), (11B) manganese (SMnC), (11C) zinc (SZnC), and (11D) copper (SCuC) of soybean plants cultivated in saline soil in the 2022 and 2023 growing seasons. The data are means ± SE (Standard Error) for three replicates. Means value that have different lower-case letter in each season are significant at *p*≤0.05 according to Duncan’s multiple range test

The results presented in Fig. 11(A-D) reveal that following the application of MgONPs as a foliar application, the levels of micronutrients in the leaves of all studied plants significantly improved. The results indicated that for SFeC and SMnC, the MgONP doses, in descending order, were ranked as follows: MgONP_1_ > MgONP_0_ > MgONP_2,_ recording 84.41 > 69.41 > 65.68 mg kg^−1^ vs. 84.03 > 68.20 > 63.95 mg kg^−1^ for SFeC and 36.55 > 36.01 > 30.40 mg kg^−1^ vs. 36.98 > 35.64 > 31.56 mg kg^−1^ for SMnC in both seasons, respectively. Similarly, for SZnC, the MgONP doses were ranked in descending order as follows: MgONP_2_ (34.09 vs. 34.36 mg kg^−1^) > MgONP_1_ (30.21 vs. 30.13 mg kg^−1^) > MgONP_0_ (28.34 vs. 26.90 mg kg^−1^) in 2022 and 2023, respectively. Meanwhile, the MgONP doses can be arranged in descending order as MgONP_1_ (15.16 vs. 14.67 mg kg^−1^) > MgONP_2_ (12.33 vs. 12.76 mg kg^−1^) > MgONP_0_ (10.03 vs. 10.75 mg kg^−1^) for SCuC in both growth seasons, respectively. The statistical analysis identified highly significant influences of MgONP doses on all studied leaf contents of micronutrients.

#### Response of mineral seed composition of salt-stressed soybean plants to the interaction between soil-applied UP and foliar-applied MgONPs

The data presented in Table 7 indicate that all studied leaf macronutrient content levels were markedly enhanced as a result of interaction between UP fertilizer rates and MgONP doses. Our investigation demonstrated that the highest values (6.92 vs. 6.96%) in SNC in both growing seasons and in SKC (2.01%) in the second season were recorded in plants fertilized with the T_41_ treatment. In addition, the maximum values in SPC (0.70%) and SMgC (0.50%) in the second season were associated with the application of T_42_. Furthermore, the plants treated with T_21_ produced the highest values in SPC (0.71%) and SCaC (0.47%) in the first season. Moreover, the use of the T_30_ treatment was the most significant for LKC (2.01%) and LMgC (0.46%) levels in the first season. Dissimilar results were obtained regarding the lowest values in seed macronutrient contents, as the plants treated with T_40_ demonstrated the minimum values in SNC (5.51%) and LPC (0.52%) in the first season, while the plants fertilized with T_10_ demonstrated the lowest mean values for SCaC (0.21%) and for SMgC (0.31%) in the second season. In addition, the application of T_12_, T_30_, and T_31_ produced the minimum values in SNC (5.55%), SPC (0.50%), and SKC (1.02%), respectively in the second season. The analysis of variance indicating that all interaction treatments had highly significant effects on all aforementioned levels of macronutrient content. According to the results listed in Table 8, the highest values in SFeC (95.02. vs. 98.88 mg kg^−1^) in both seasons and the highest levels of SMnC (50.00 mg kg^−1^) in the 2022 season and of SCuC (19.75 mg kg^−1^) in the 2023 season were achieved in the plants fertilized with T_41_. Furthermore, the maximum SZnC (38.91 mg kg^−1^) and SCuC (19.87 mg kg^−1^) was recorded during the first season in plants treated with T_22_. The application of T_32_ was the most impactful on SMnC (49.53 mg kg^−1^) and SZnC (42.24 mg kg^−1^) in the second season. On the other hand, the lowest values in SMnC (18.57 vs. 16.87 mg kg⁻¹) and SCuC (5.00 vs. 5.89 mg kg⁻¹) in 2022 and 2023, respectively were obtained in plants treated with T₁₂ and T₂₀. Meanwhile, the plants fertilized with T_42_ demonstrated the lowest value in SFeC (55.33 mg kg⁻¹) in the second season and in SZnC (20.72 mg kg⁻¹) in the first season. Moreover, the application of T_30_ and T₁₀ produced the lowest values in SFeC (54.49 mg kg⁻¹) in the first season and in SZnC (20.17 mg kg⁻¹) in the second season.

**Table 7:** Impact of the interaction between urea-phosphate (UP) as a soil application and magnesium oxide nanoparticles (MgONPs) as a foliar nourishment on the seed macronutrient contents of soybean plants cultivated in saline-sodic soil during two consecutive seasons (2022 and 2023)

**UP**	**MgONP**	**Symbol**	**SNC**	**SPC**	**SKC**	**SCaC**	**SMgC**
			**(%)**				
2022 growth season							
UP_1_	MgONP_0_	T_10_	6.91±0.10a	0.57±0.10de	1.96±0.20b	0.41±0.20cd	0.36±0.30d
	MgONP_1_	T_11_	6.59±0.20c	0.59±0.20d	1.02±0.30h	0.36±0.10ef	0.39±0.20d
	MgONP_2_	T_12_	6.36±0.20d	0.67±0.10 a-c	1.96±0.30b	0.43±0.10bc	0.44±0.10ab
UP_2_	MgONP_0_	T_20_	5.47±0.10e	0.56±0.30de	1.07±0.20g	0.33±0.10f	0.30±0.20e
	MgONP_1_	T_21_	5.70±0.10e	0.71a±0.20a	1.89±0.10c	0.47±0.10a	0.33±0.20e
	MgONP_2_	T_22_	5.55±0.10f	0.64c±0.10c	1.53±0.30ef	0.37±0.20de	0.43±0.30b
UP_3_	MgONP_0_	T_30_	6.84±0.20b	0.58d±0.20d	2.01±0.20a	0.36±0.20ef	0.46±0.20a
	MgONP_1_	T_31_	6.41±0.30d	0.65±0.10bc	1.08±0.20g	0.46±0.10ab	0.42±0.10bc
	MgONP_2_	T_32_	6.57±0.10c	0.67 ±0.10a-c	1.56±0.10e	0.35±0.30ef	0.42±0.10bc
UP_4_	MgONP_0_	T_40_	5.51±0.10f	0.52±0.20e	1.98±0.20ab	0.23g±0.2	0.32±0.30e
	MgONP_1_	T_41_	6.92±0.20a	0.70±0.10ab	1.50±0.30f	0.20g±0.2	0.42±0.10bc
	MgONP_2_	T_42_	6.78±0.20b	0.59±0.20d	1.63±0.10d	0.44a-c±0.1	0.41±0.20bc
*p*-value							
UP (A)			0.000^**^	0.041^*^	0.000^**^	0.000^**^	0.002^**^
MgONP (B)			0.000^**^	0.000^**^	0.000^**^	0.000^**^	0.000^**^
UP (A) x MgONP (B)			0.000^**^	0.000^**^	0.000^**^	0.000^**^	0.000^**^
2023 growth season							
UP_1_	MgONP_0_	T_10_	5.68±0.10h	0.51±0.10d	1.96±0.20b	0.21±0.10g	0.31±0.20f
	MgONP_1_	T_11_	6.27±0.20e	0.66 ±0.20a-c	1.76±0.10d	0.22±0.20fg	0.34±0.20c-f
	MgONP_2_	T_12_	5.55±0.20i	0.62±0.10c	1.63±0.10e	0.25±0.10e-g	0.36±0.20cd
UP_2_	MgONP_0_	T_20_	6.87±0.10b	0.52±0.20d	1.42±0.30f	0.25±0.20ef	0.33±0.10d-f
	MgONP_1_	T_21_	5.89±0.10fg	0.52±0.20d	1.93±0.20b	0.21±0.20fg	0.32±0.10ef
	MgONP_2_	T_22_	6.44±0.10d	0.64±0.20bc	1.85±0.10c	0.37±0.30b	0.35±0.20c-e
UP_3_	MgONP_0_	T_30_	5.93±0.10f	0.50±0.20d	1.06±0.10h	0.26±0.20de	0.33±0.20d-f
	MgONP_1_	T_31_	6.29±0.20e	0.67±0.10ab	1.02±0.20h	0.30±0.30cd	0.50±0.10a
	MgONP_2_	T_32_	6.32±0.20e	0.66 ±0.20a-c	1.38±0.10f	0.46±0.30e	0.31±0.10f
UP_4_	MgONP_0_	T_40_	5.82±0.20g	0.51d±0.3	1.83±0.20c	0.33±0.10c	0.40±0.20b
	MgONP_1_	T_41_	6.96±0.10a	0.53d±0.2	2.01±0.30a	0.40±0.10b	0.37±0.20bc
	MgONP_2_	T_42_	6.62±0.10c	0.70a±0.2	1.16±0.10g	0.23±0.10e-g	0.50±0.20a
*p*-value							
UP (A)			0.000^**^	0.075^ns^	0.000^**^	0.000^**^	0.000^**^
MgONP (B)			0.000^**^	0.000^**^	0.000^**^	0.000^**^	0.000^**^
UP (A) x MgONP (B)			0.000^**^	0.000^**^	0.000^**^	0.000^**^	0.000^**^

The data are means ± SE (Standard Error) for three replicates. The mean values with different lowercase letters during each season are significant at *p *≤ 0.05 according to Duncan’s multiple range test. SNC, SPC, SKC, SCaC, and SMgC indicate the leaf nitrogen, phosphorus, potassium, calcium, and magnesium contents, respectively. UP_1_, UP_2_, UP_3_, and UP_4_ represent urea-phosphate at 85.0, 107.0, 127.0 and 150.0 kg ha^−1^, respectively. MgONP_0_, MgONP_1_, and MgONP_2_ represent magnesium oxide nanoparticles at 0.00, 50.0, and 100.0 mg L^−1^, respectively

**Table 8:** Impact of the interaction between urea-phosphate (UP) as a soil application and magnesium oxide nanoparticles (MgONPs) as a foliar nourishment on the seed micronutrient contents of soybean plants cultivated in saline-sodic soil during two consecutive seasons (2022 and 2023)

UP	MgONP	Symbol	SFeC	SMnC	SZnC	SCuC
			(mg kg^−1^)			
2022 growth season						
UP_1_	MgONP_0_	T_10_	74.39±0.40c	39.37±0.50c	21.11±0.30d	13.40±0.30b-d
	MgONP_1_	T_11_	73.20±0.30c	21.75±0.30f	34.24±0.40a-c	10.45±0.20e-g
	MgONP_2_	T_12_	60.14±0.30de	18.57±0.50f	38.33±0.40ab	8.30±0.30 g
UP_2_	MgONP_0_	T_20_	87.08±0.30b	37.89±0.30c	35.70±0.40a-c	5.00±0.40 h
	MgONP_1_	T_21_	83.91±0.50b	47.54±0.30ab	34.72±0.40a-c	14.55±0.30bc
	MgONP_2_	T_22_	64.19±0.20d	22.38±0.20f	38.91±0.50a	19.87±0.30a
UP_3_	MgONP_0_	T_30_	54.49±0.40f	22.91±0.40ef	23.94±0.50d	12.36±0.20c-e
	MgONP_1_	T_31_	85.49±0.30b	26.91±0.40e	30.19±0.20c	15.83±0.40b
	MgONP_2_	T_32_	82.67±0.30b	47.23±0.30ab	38.40±0.30ab	11.20±0.40d-f
UP_4_	MgONP_0_	T_40_	61.68±0.30d	43.89±0.40b	32.60±0.30bc	9.37±0.30 fg
	MgONP_1_	T_41_	95.02±0.50a	50.00±0.40a	21.68±0.30d	19.80±0.30a
	MgONP_2_	T_42_	55.73±0.40ef	33.45±0.30d	20.72±0.40d	9.96±0.20e-g
*p*-value						
UP (A)			0.000^**^	0.000^**^	0.000^**^	0.002^**^
MgONP (B)			0.000^**^	0.000^**^	0.001^**^	0.000^**^
UP (A) x MgONP (B)			0.000^**^	0.000^**^	0.000^**^	0.000^**^
2023 growth season						
UP_1_	MgONP_0_	T_10_	67.80±0.30 cd	38.49±0.30c	20.17±0.30 h	13.59±0.40 cd
	MgONP_1_	T_11_	70.16±0.50c	19.73±0.40ef	30.52±0.30de	10.76±0.40e
	MgONP_2_	T_12_	57.83±0.30ef	16.87±0.30f	36.17±0.30bc	8.59±0.30f
UP_2_	MgONP_0_	T_20_	85.90±0.20b	37.30±0.30c	30.03±0.50de	5.87±0.40 g
	MgONP_1_	T_21_	84.55±0.30b	49.02±0.30ab	36.76±0.40b	13.33±0.40d
	MgONP_2_	T_22_	62.00±0.30e	20.47±0.40ef	37.69±0.10ab	19.07±0.20a
UP_3_	MgONP_0_	T_30_	56.13±0.50f	23.47±0.20e	25.93±0.20e-g	14.72±0.10bc
	MgONP_1_	T_31_	82.53±0.30b	31.16±0.40d	29.01±0.40d-f	14.85±0.40b
	MgONP_2_	T_32_	80.63±0.40b	49.53±0.40a	42.24±0.30a	11.62±0.50e
UP_4_	MgONP_0_	T_40_	62.97±0.30de	43.28±0.30bc	31.49±0.30 cd	8.82±0.40f
	MgONP_1_	T_41_	98.88±0.30a	48.01±0.40ab	24.23±0.50f-h	19.75±0.30a
	MgONP_2_	T_42_	55.33±0.30f	39.36±0.40c	21.34±0.40gh	11.78±0.30e
*p*-value						
UP (A)			0.000^**^	0.000^**^	0.001^**^	0.000^**^
MgONP (B)			0.000^**^	0.002^**^	0.000^**^	0.000^**^
UP (A) x MgONP (B)			0.000^**^	0.000^**^	0.000^**^	0.000^**^

The data are means ± SE (Standard Error) for three replicates. The mean values with different lowercase letters during each season are significant at *p* ≤ 0.05 according to Duncan’s multiple range test. SFeC, SMnC, SZnC, and SCuC indicate the seed iron, manganese, zinc, and copper contents, respectively. UP_1_, UP_2_, UP_3_, and UP_4_ represent urea-phosphate at 85.0, 107.0, 127.0 and 150.0 kg ha^−1^, respectively. MgONP_0_, MgONP_1_, and MgONP_2_ represent magnesium oxide nanoparticles at 0.00, 50.0, and 100.0 mg L^−1^, respectively

### Yield and its attributes

#### Response of yield and its attributes of salt-stressed soybean plants to UP soil application

**Fig. 12:**
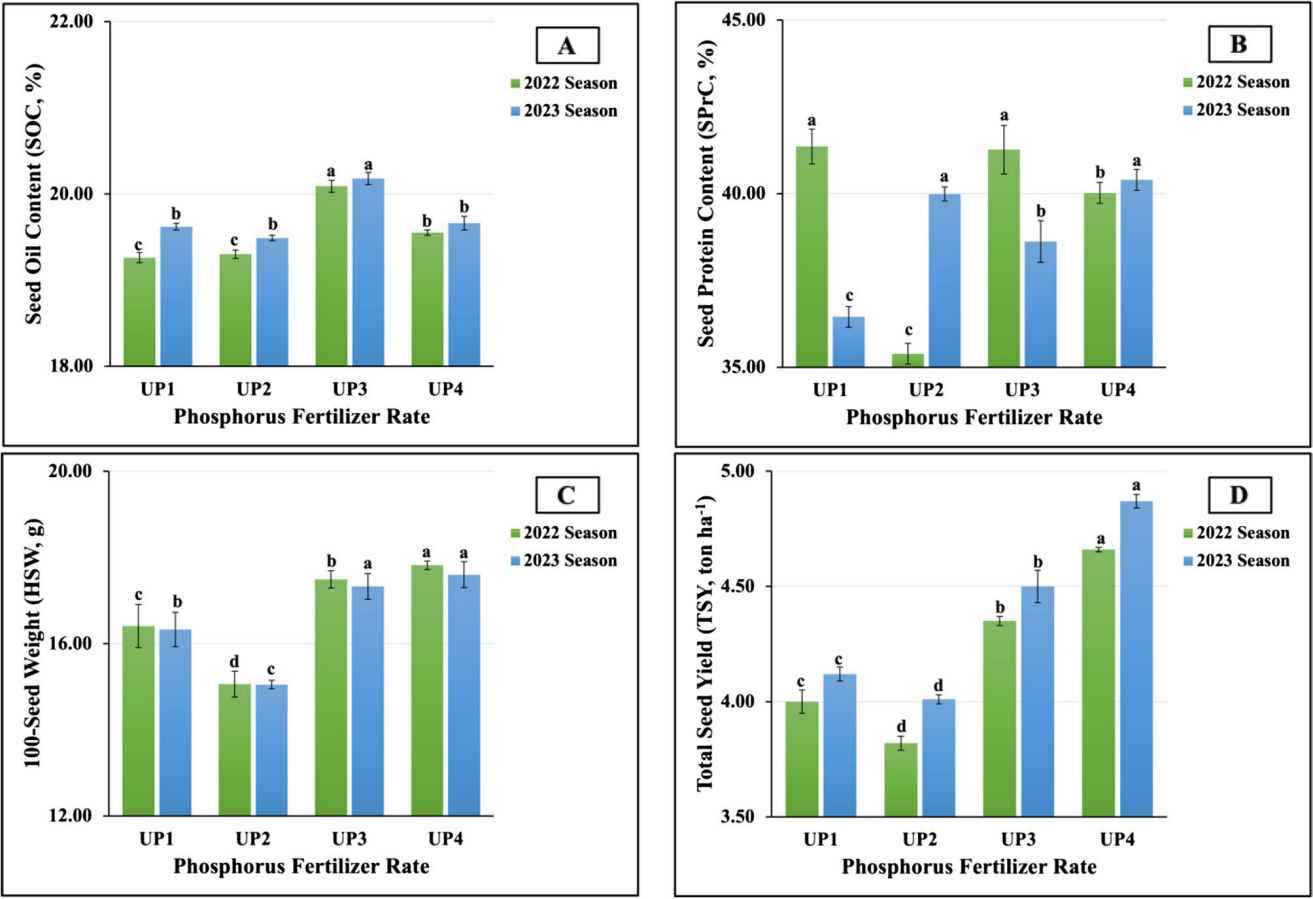
**A**-**D **The individual impact of urea-phosphate rates (UPs) on (12A) SOC, (12B) SPrC, (12C) HSW, and (12D) TSY of soybean plants cultivated in saline soil in the 2022 and 2023 growing seasons. The data are means ± SE (Standard Error) for three replicates. Means value that have different lower-case letter in each season are significant at *p*≤0.05 according to Duncan’s multiple range test

The results obtained from the ANOVA clearly indicated that all UP treatments had highly significant effects on 100-seed weight (HSW), seed oil content (SOC), seed protein content (SPC), and total seed yield (TSY) in both growth seasons. As visually evident in Fig. 12(A-D), the highest values in SOC (20.09 vs. 20,18%) were produced in plants fertilized with UP_3_ in both growing seasons. Meanwhile, the maximum values in HSW (17.82 vs. 17.60 g) and TSY (4.66 vs. 4.87ton ha^−1^) in the 2022 and 2023 seasons, respectively were obtained in plants treated with UP_4_. Although UP_4_ had a profound impact on SPrC in the second season, the use of UP_1_ produced the highest value in the first season. Similarly, the lowest values in HSW (15.06 vs. 15.08 g) and TSY (3.82 vs. 4.01ton ha^−1^) were obtained in plants treated with UP_2_, while the application of UP_1_ produced the lowest SOC (19.26%) in the first season and the lowest SPC (36.45%) in the second season. Simultaneously, the use of UP_2_ produced the minimum values in SPrC (35.39%) in the first season and the minimum value in SOC (19.49%) in the second season.

#### Response of yield and its attributes of salt-stressed soybean plants to MgONPs foliar application

**Fig. 13:**
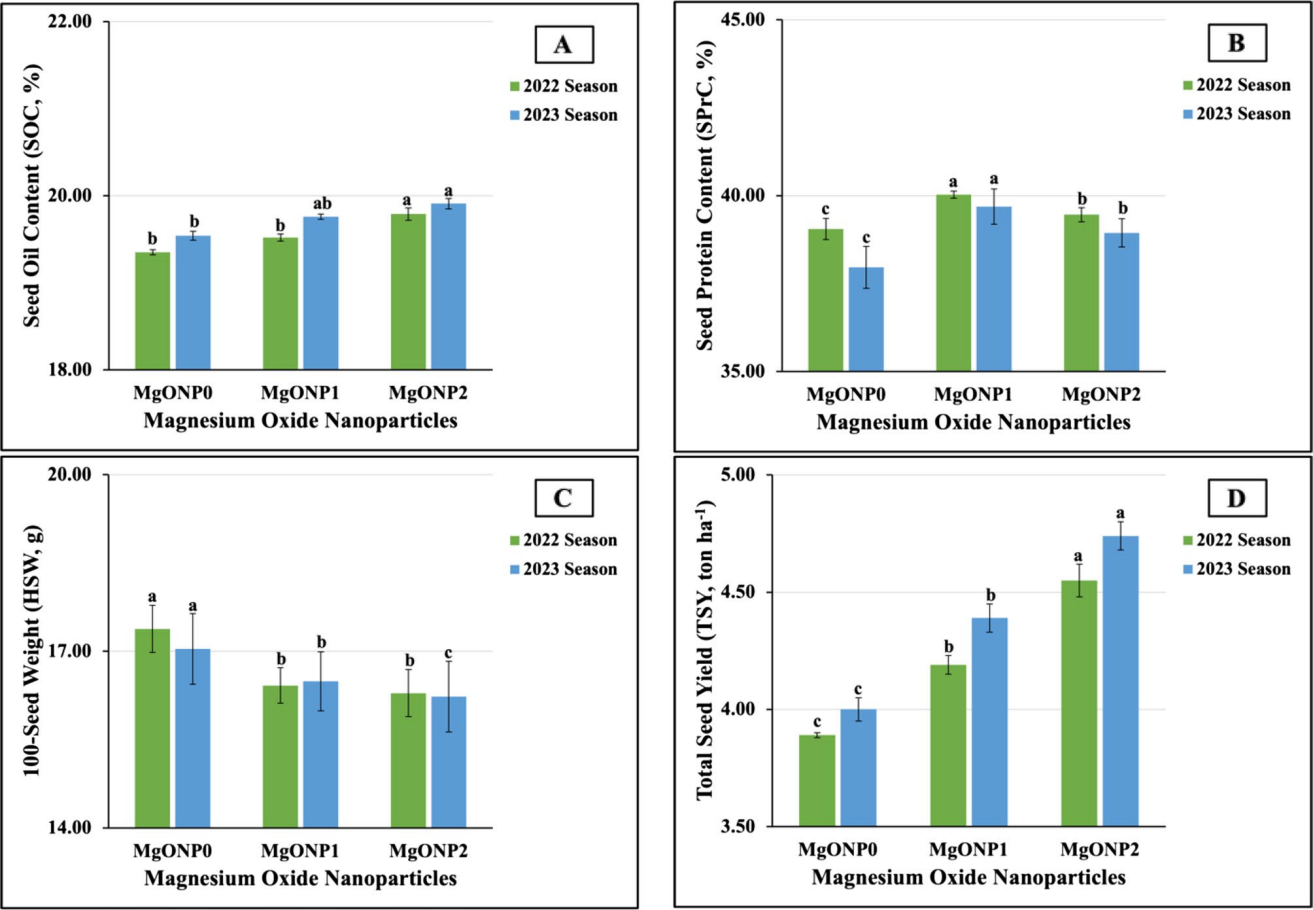
**A**-**D **The individual impact of magnesium oxide nanoparticles doses (MgONPs) on (13A) SOC, (13B) SPrC, (13C) HSW, and (13D) TSY of soybean plants cultivated in saline soil in the 2022 and 2023 growing seasons. The data are means ± SE (Standard Error) for three replicates. Means value that have different lower-case letter in each season are significant at *p*≤0.05 according to Duncan’s multiple range test

The results obtained from the statistical analysis indicated that MgONPs treatments significantly (at *p* ≤ 0.01) affected yield and its components. As graphically demonstrated in Fig. 13(A-D), the plants foliarly sprayed with MgONP_2_ produced the maximum values in SOC (19.79 vs. 19.91%) and TSY (4.55 vs. 4.74ton ha^−1^) in the first and second seasons. Furthermore, the highest SPrC values (40.03 vs. 39.69%) were produced in plants treated with MgONP_1_. Moreover, the highest values in HSW (17.38 vs. 17.04 g) were recorded in untreated plants in both growth seasons. On the other hand, MgONP_2_ treatment was the least influential on HSW, producing HSW levels of 16.29 vs. 16.23 g in the two growing seasons. Meanwhile, the lowest values in SPrC (39.05 vs. 37.96%), TSY (3.89 vs. 4.00ton ha^−1^), and SOC (19.35 vs. 19.54%) in the 2022 and 2023 seasons, respectively were obtained in untreated plants.

#### Response of yield and its attributes of salt-stressed soybean plants to the interaction between soil-applied UP and foliar-applied MgONPs

The data explored in Table 9 indicate that plants treated with both UP as a soil application and MgONPs as a foliar spray, irrespective of their doses, markedly outperformed the control treatment, in terms of enhanced productivity and yield-related attributes, although the ANOVA data revealed that all treatments had significant influences (at *p* ≤ 0.01) on the HSW, SOC, and SPC. Conversely, there were no significant impacts on TSY in both growth seasons, respectively. We found that the co-application of UP_4_ and MgONP_1_ (T_41_) was the superior treatment; it produced the maximum values for HSW (18.77 vs. 18.53 g) and for SPrC (43.25 vs. 43.48%) in both growing seasons. Meanwhile, the T_42_ treatment was the most impactful on TSY, producing the highest values (5.00 vs. 5.19ton ha^−1^) in both seasons, respectively. Dissimilar results were obtained for SOC; however, T_31_ and T_40_ produced the best values (20.75 vs. 21.02%) in the 2022 and 2023 seasons, respectively. Conversely, the lowest values in HSW (13.27 vs. 13.00 g) and TSY (3.54 vs. 3.62 tan ha^−1^) in the first and second seasons, respectively were produced in plants treated with T_22_ and T_20_. Interestingly, the application of T_40_ and T_12_ for SPrC and T_11_ and T_41_ for SOC were the least impactful, producing the minimum values for SPrC (34.42 vs. 34.67%) and SOC (18.40 vs. 18.56%) in the two growing seasons, respectively.

**Table 9:** Impact of the interaction between urea-phosphate (UP) as a soil application and magnesium oxide nanoparticles (MgONPs) as a foliar nourishment on the yield and its components of soybean plants cultivated in saline-sodic soil during two consecutive seasons (2022 and 2023)

UP	MgONPs	Symbol	SOC	SPrC	HSW	TSY
			(%)		(g)	(ton ha^−1^)
2022 growth season						
UP_1_	MgONP_0_	T_10_	19.14±0.03 cd	43.19±0.10a	16.97±0.40d	3.54±0.02f
	MgONP_1_	T_11_	18.40±0.04e	41.17±0.20c	14.63±0.20 g	3.97±0.01e
	MgONP_2_	T_12_	20.23±0.07b	39.73±0.20d	17.63±0.20c	4.34±0.01c
UP_2_	MgONP_0_	T_20_	18.78±0.03de	35.85±0.30e	16.73±0.30d	3.54±0.03 g
	MgONP_1_	T_21_	20.13±0.04b	35.65±0.20e	15.17±0.30f	3.79±0.02f
	MgONP_2_	T_22_	18.99±0.02 cd	34.67±0.20f	13.27±0.20 h	4.14±0.01d
UP_3_	MgONP_0_	T_30_	18.95±0.04 cd	42.73±0.20b	17.17±0.30 cd	4.02±0.02e
	MgONP_1_	T_31_	20.75±0.03a	40.04±0.10d	17.10±0.10 cd	4.34±0.02c
	MgONP_2_	T_32_	20.58±0.03ab	41.04±0.10c	18.20±0.20b	4.69±0.01b
UP_4_	MgONP_0_	T_40_	20.52±0.02ab	34.42±0.20f	18.63±0.20ab	4.3±0.04c
	MgONP_1_	T_41_	18.78±0.03de	43.25±0.20a	18.77±0.30a	4.66±0.02b
	MgONP_2_	T_42_	19.35±0.02c	42.40±0.10b	16.07±0.20e	5.00±0.02a
*p*-value						
UP (A)			0.000^**^	0.000^**^	0.000^**^	0.000^**^
MgONP (B)			0.002^**^	0.000^**^	0.000^**^	0.000^**^
UP (A) x MgONP (B)			0.000^**^	0.000^**^	0.000^**^	0.887^ns^
2023 growth season						
UP_1_	MgONP_0_	T_10_	19.35±0.03bc	35.52±0.30 h	16.00±0.20d	3.71±0.03e
	MgONP_1_	T_11_	19.80±0.01b-d	39.17±0.20e	14.57±0.20 g	4.15±0.02d
	MgONP_2_	T_12_	20.60±0.02a	34.67±0.10i	17.83±0.30b	4.51±0.02c
UP_2_	MgONP_0_	T_20_	18.82±0.03 cd	42.92±0.20b	16.67±0.30d	3.62±0.02e
	MgONP_1_	T_21_	20.67±0.03a	36.79±0.10 fg	15.47±0.40f	4.02±0.01d
	MgONP_2_	T_22_	18.98±0.04b-d	40.25±0.20d	13.10±0.20 h	4.39±0.01c
UP_3_	MgONP_0_	T_30_	18.97±0.01b-d	37.04±0.30f	16.83±0.30d	4.11±0.02d
	MgONP_1_	T_31_	20.93±0.02a	39.33±0.20e	17.40±0.20c	4.52±0.04c
	MgONP_2_	T_32_	20.66±0.03a	39.48±0.40e	17.77±0.40bc	4.87±0.03b
UP_4_	MgONP_0_	T_40_	21.02±0.03a	36.38±0.20 g	18.07±0.20b	4.56±0.01c
	MgONP_1_	T_41_	18.56±0.02d	43.48±0.10a	18.53±0.20a	4.87±0.01b
	MgONP_2_	T_42_	19.39±0.02b	41.35±0.20c	16.20±0.30e	5.19±0.02a
*p*-value						
UP (A)			0.006^**^	0.000^**^	0.000^**^	0.000^**^
MgONP (B)			0.024^*^	0.000^**^	0.000^**^	0.000^**^
UP (A) x MgONP (B)			0.000^**^	0.000^**^	0.000^**^	0.882^ns^

The data are means ± SE (Standard Error) for three replicates. The mean values with different lowercase letters during each season are significant at *p* ≤ 0.05 according to Duncan’s multiple range test. SOC, and SPrC indicate the seed oil and protein contents, respectively. HSW, TSY indicate 100-Seed weight and total seed yield, respectively. UP_1_, UP_2_, UP_3_, and UP_4_ represent urea-phosphate at 85.0, 107.0, 127.0 and 150.0 kg ha^−1^, respectively. MgONP_0_, MgONP_1_, and MgONP_2_ represent magnesium oxide nanoparticles at 0.00, 50.0, and 100.0 mg L^−1^, respectively

#### Principal component, pearson’s correlation, and stepwise multiple regression analyses

**Fig. 14:**
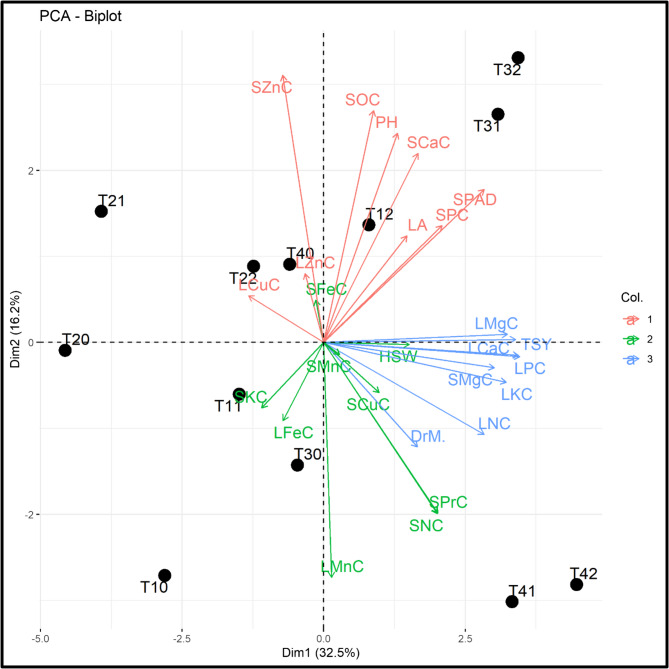
Principal component analysis (PCA) of applied urea–phosphate (UP) and magnesium oxide nanoparticle (MgONP) treatments and studied parameters. Each black dot denotes a treatment. SPAD, PH, LA, and DrM indicate the relative chlorophyll content, plant height, leaf area, and dry matter percentage, respectively. LNC, LPC, LKC, LCaC, LMgC, LFeC, LMnc, LZnC, and LCuC indicate the leaf nitrogen, phosphorus, potassium, calcium, magnesium, iron, manganese, zinc, and copper contents, respectively. SNC, SPC, SKC, SCaC, SMgC, SFeC, SMnC, SZnC, and SCuC indicate the seed nitrogen, phosphorus, potassium, calcium, magnesium, iron, manganese, zinc, and copper contents, respectively. SOC, SPrC, and TSY indicate the seed oil content, protein content, and total seed yield, respectively. Values are based on averages of two consecutive seasons (2022 and 2023). T_10_, T_11_, and T_12_ represent the UP applied at 85.0 kg ha^−1^ with three doses of MgONPs: 0.00, 50.0, and 100.0 mg L^−1^, respectively. T_20_, T_21_, and T_22_ represent the UP applied at 107.0 kg ha^−1^ with three doses of MgONPs: 0.00, 50.0, and 100.0 mg L^−1^, respectively. T_30_, T_31_, and T_32_ represent the UP applied at 127.0 kg ha^−1^ with three doses of MgONPs: 0.00, 50.0, and 100.0 mg L^−1^, respectively. T_40_, T_41_, and T_42_ represent the UP applied at 85.0 kg ha^−1^ with three doses of MgONPs: 0.00, 50.0, and 100.0 mg L^−1^, respectively

Principal component, Pearson’s correlation, and stepwise multiple regression analyses were performed on the physiological–growth attributes, leaf nutrient contents, and yield- and quality-related parameters of soybean plants cultivated in saline–sodic soil. Principal component analysis (PCA) was performed to evaluate the relations between the UP x MgNP interaction treatments and the abovementioned characteristics. As shown in Fig. 14, the PCA indicated that the first two main components, Dim 1 and Dim 2 (PCA-diminution 1 and -diminution 2, respectively), accounted for 48.7% of the total variation. PC1 interpreted 32.5% of the variation. The nearby vectors of the measured parameters presented a positive correlation with one another. However, the SPAD readings, PH, LA, LMgC, SPC, SCaC, SOC, and TSY fell under the same group, while the LNC, LPC, LKC, LCaC, LMnC, SNC, SMgC, SPrC, and HSW were in a separate group.

The PCA biplot in Fig. 13 shows that the SPAD, PH, LA, LMgC, SPC, SCaC, and SOC were improved by T12, T31, and T32. Moreover, the LNC, LPC, LKC, LCaC, LMnC, HSW, DrM, SNC, SMnC, and SPrC were also enhanced by T41 and T42. Therefore, the application of UP and MgONP interaction plays a crucial role in promoting most of the traits associated with the nutritional status, yield, and their components.

The results provided in Table 10 indicate the correlations of various physiological attributes that were determined (SPAD reading, LA, PH, and DrM%) and of the nutrient content in leaves (LNC, LPC, LKC, LCaC, LMgC, LFeC, LMnC, LZnC, and LCuC), with the TSY and SOC in both growth seasons, respectively. Our results revealed that SPAD readings correlated (*r* = 0.419^*^ vs. 0.589^**^ in the first and second seasons, respectively) with TSY and (*r* = 0.437^**^ vs. 0.349^*^) with SOC in the first and second seasons, respectively. The influence of PH was found to be more correlated with SOC, with correlation values of *r* = 0.354^*^ and 0.368 in the 2022 and 2023 seasons, respectively. Similarly, TSY had highly significant positive correlations with LNC (*r* = 0.351^*^ vs. 0.951^*^), LPC (*r* = 0.953^**^ vs. 0.934^**^), LKC (*r* = 0.642^**^ vs. 0.826^**^), LCaC (*r* = 0.801^**^ vs. 0.788^**^), and LMgC (*r* = 0.711^**^ vs. 0.697^**^) in 2022 and 2023, respectively. A highly significant negative correlation of SOC was found with LMgC (*r* = −0.432^**^ vs. −0.461^**^ in 2022 and 2023, respectively).

**Table 10:** Pearson’s correlation coefficient between total seed yield (TSY) and seed oil content (SOC) with 13 selected attributes of soybean plants fertilized with urea-phosphate (UP) as a soil application and magnesium oxide nanoparticles (MgONPs) as a foliar nourishment under saline-sodic soil during two consecutive seasons (2022 and 2023)

Character	TSY		SOC		TSY		SOC	
	Pearson *r*	Probabilities	Pearson *r*	Probabilities	Pearson *r*	Probabilities	Pearson *r*	Probabilities
	2022 growth season				2023 growth season			
Plants’ physiological attributes								
SPAD reading	0.419*	0.011	0.437**	0.008	0.589**	0.000	0.349*	0.037
PH	0.143	0.404	0.354*	0.034	0.138	0.423	0.368	0.531
LA	0.088	0.610	0.102	0.553	0.122	0.478	0.194	0.258
DrM%	0.298	0.078	0.056	0.745	0.252	0.137	−0.048	0.782
Leaf nutrient contents								
LNC	0.351*	0.036	−0.158	0.358	0.951**	0.000	0.194	0.257
LPC	0.953**	0.000	0.298	0.077	0.934**	0.000	0.186	0.277
LKC	0.642**	0.000	0.203	0.235	0.826**	0.000	0.208	0.223
LCaC	0.801**	0.000	0.239	0.160	0.788**	0.000	0.197	0.249
LMgC	0.711**	0.000	0.136	0.428	0.697**	0.000	0.167	0.331
LFeC	−0.142	0.410	−0.278	0.101	−0.272	0.108	−0.233	0.172
LMnC	0.058	0.328	−0.432**	0.009	0.019	0.914	−0.461**	0.005
LZnC	−0.168	0.328	0.175	0.308	−0.098	0.569	0.236	0.166
LCuC	−0.180	0.293	−0.161	0.347	−0.144	0.402	−0.069	0.687
SOC	0.297	0.079	1	---	0.209	0.220	1	---

** = *p* ≤ 0.01 and * = *p* ≤ 0.01. TSY and SOC indicate the total seed yield and seed oil content, respectively. SPAD reading, PH, LA and DrM% indicate the Soil-Plant-Analysis Development, plant height, leaf area, and leaf dry matter percentage, respectively. LNC, LPC, LKC, LcaC, LMgC, LfeC, LMnC, LZnC, and LcuC indicate leaf nitrogen, phosphorus, potassium, calcium, magnesium, iron, manganese, zinc and copper contents, respectively

As observed in Table 11, stepwise regression analysis clearly identified the relationship between TSY and SOC as a response variable with physiological attributes (SPAD reading and LA), leaves’ nutrient contents (LNC, LPC, LKC, LCaC, and LMnC), and yield-related attribute as predictor variables. The obtained results revealed that model 3 and model 2 were the most suitable in the 2022 and 2023 growth seasons, respectively. However, these models had high adjusted R^2^ 0.931 (0.968) and 0.924 (0.964) and the lowest SEE (0.113 and 0.129). These results demonstrate that 93.1% of variations in TSY occurred because of variations in the combination of LPC, LA, and LCaC (TSY = 2.014 LPC + 4.842 LA + 0.004 LCaC in the first season). According to model 2, 92.4% of variations in TSY were due to variations in the combination of LNC and LKC (TSY = 0.627 LNC + 1.990 LKC). With regard to SOC, model 3 in the 2022 season and model 2 in the 2023 season were the best models owing to their maximum adjusted R^2^, which was recorded as 0.344 (0.618) in the first season and 0.278 (0.565) in the second season, and due to achieving the lowest SEE, which was recorded as 0.673 and 0.808 in the 2022 and 2023 season, respectively. The adjusted R^2^ demonstrated 34.4% and 27.8% of variations in the combination of SPAD readings, LMnC, and SOC (16.695 SPAD reading + 0.082 LMnC) in the first season and 27.8% of variations in the combinations of LMnC, HSW, and SOC (17.386 LMnC − 0.016 HSW) in the second season.

**Table 11:** Proportional contribution in predicting total seed yield (TSY) and seed oil content (SOC) using Stepwise linear regression for salt-stressed soybean plants fertilized with urea-phosphate (UP) as a soil application and magnesium oxide nanoparticles (MgONPs) as a foliar nourishment under saline-sodic soil during two consecutive seasons (2022 and 2023)

Model	Entered variable	Intercept	b_1_	b_2_	b_3_	*r*	*R* ^2^	Adj. *R*^2^	SEE
2022 growth season									
TSY									
1	LPC	2.121**	5.568			0.953^a^	0.909	0.906	0.132
2	LA	2.256*	5.712	−0.003		0.960^b^	0.922	0.917	0.124
3	LCaC	2.014**	4.842	−0.004	1.078	0.968^c^	0.937	0.931	0.113
SOC									
1	SPAD reading	15.888	0.081			0.437^a^	0.191	0.168	0.759
2	LMnC	16.695	0.082	−0.014		0.618^b^	0.381	0.344	0.673
2023 growth season									
TSY									
1	LNC	−0.332	1.405			0.951^a^	0.904	0.901	0.148
2	LKC	−0.627	1.990	−0.610		0.964^b^	0.929	0.924	0.129
SOC									
1	LMnC	20.778	−0.017			0.461^a^	0.212	0.189	0.857
2	HSW	17.386	−0.016	0.202		0.565^b^	0.320	0.278	0.808

** = *p* ≤ 0.01 and * = *p* ≤ 0.01. LPC, LA, LCaC, LMnC, LNC and LKC indicate the leaf phosphorus, calcium, manganese, nitrogen and potassium contents, respectively. LA and SPAD reading indicate the leaf area, and Soil-Plant-Analysis development, respectively. HSW 100-seed weight

## Discussion

### Impact of urea–phosphate as a soil application on salt-stressed soybean plants

Phosphorus (P) is known to be one of the three most essential macronutrients, alongside nitrogen [54–56]. Although P is abundant in the Lithosphere, the amount available to and absorbed by plants is very small [57]. Notably, in our research, improvements in plant growth and development were closely associated with the application of a urea–phosphate (UP) fertilizer at rates of 127.0 and 150.0 kg ha^−1^. The application of high amounts of P possibly led to nutritional balance [58]. On the contrary, the application of low amounts of P (85.0 kg ha^−1^) increased the plant dry matter percentage (DrM%). These findings could be attributed to the application of high rates of UP decreasing the length, root surface, and volume of roots [58]. These results were in accordance with those of a previous study [17] that reported no significant difference between high and low P rates on DrM%. Numerous studies have been conducted on the effect of P application on growth–physiological attributes under abiotic stresses [59–61]. However, P plays a significant role in enhancing physiological functions and increasing the plants’ tolerance to abiotic stress [62, 63]. Other studies [64–66] have reported that the ability of plants to overcome abiotic stress, such as salinity, can be improved by modulating their phosphorus metabolism. UP showed highly significant influences on the uptake of other macronutrients such as nitrogen (LNC), potassium (LKC), calcium (LCaC), and magnesium (LMgC) in our field study. These findings were in a line with the previous results, however Mussarat et al. 2021 [67] noted that maize growth significantly enhanced by improving nutrient solubilizing and availability. Regarding the LNC, our findings were consistent with those from most previous studies [68, 69], as a high P supply level can promote nitrogenase activity, especially in soybean plants. These findings were similar to those from the investigations of [70], in which the nitrogen fixation of nodules was reported to be closely correlated with low phosphorus levels. This may be attributed to the profound effect of P in the formation of ATPase enzymes and energy transfer and their regulation for plants under salinity conditions [71]. Another explanation was reported in [72], which documented that an increase in phosphorus fertilizer can balance nutrients and promote plant nutrient uptake by enhancing their availability. Furthermore, a high UP supply plays a significant role in reducing soil pH, especially in alkaline soil (pH>7.0). Accordingly, this reduction in pH around the rhizosphere could be the main factor in the nutrients released by microorganisms [73]. Furthermore, the work of [74, 75] documented that decreased pH prevents the reaction of phosphorus with calcium during the formation of insoluble compounds such as hydroxyapatite.

Protein and oil are important constituents of soybean seeds. Numerous studies have emphasized that their increase or decrease is closely related to P fertilizer rates, soil moisture content, and varietal characteristics, as reported by Taliman et al. [17] study. In a previous study, Yi et al. [76] found that SOC was decreased and SPrC was increased, with higher P fertilizer rates. While, there were strong positive between P rate and both SOC and SPrC [77]. Significant variations were also observed in the seed contents of K, Ca, and Mg (Fig. 8), which may be associated with differences in phytate content arising from variations in P fertilizer rate [17]. These findings support previous research indicating that both Ca and Mg bind with phytic acid, thereby reducing its buffering effect [78]. On the opposite, decreases in seed micronutrient contents, especially Fe, Zn, and Cu were associated with the application of UP_2_ (Fig. 9). Earlier studies by Sanchez-Rodrigues et al. [79] and Naeem et al. [80] reported that SZnC was negatively affected with high P fertilizer rates. These results were confirmed by Zhang et al. [81], who reported a significant decline in SZnC as a result of P fertilization, owing to its profound effect on the bioavailability of these nutrients [82].

In the present research, the application of UP_4_ was the best treatment for enhancing the TSY in saline-sodic soil. These results were consistent with previous studies that indicated that the increasing P fertilizers might be improved the activity of N metabolism related enzymes and enhanced N metabolism [38, 58].

### Impact of magnesium oxide nanoparticles as a foliar application on salt-stressed soybean

In this investigation, we documented both the complicated and unpredictable impacts of magnesium oxide nanoparticles (MgONPs) on soybean plants cultivated under saline conditions. As presented in Fig. 1(A), the SPAD readings increased in the soybean plants that were foliarly treated with MgONP_2_ compared with those of the untreated plants. Mg is a crucial element for chlorophyll formation and is therefore essential to the photosynthesis of plants [82, 83]. In addition, Mg is an important nutrient in the utilization of photo-assimilates, photophosphorylation, and photo-oxidation, as well as in the generation of reactive oxygen species [84]. In other words, Mg participates in many physiological and biochemical reactions that are mainly related to photosynthesis and photo-assimilation [20]. Similarly, the highest plant height (PH) and plant dry matter percentage (DrM%) values were obtained in the plants fertilized with MgONPs. Very recently, the authors of [85] clearly illustrated that MgONPs provide profound growth–physiological effects, such as improved photosynthesis abilities and tissue shoot growth. Furthermore, MgONPs have the greatest influence on plant growth out of all the other types of nanomaterials [39]. In other words, these impacts could be attributed to the profound role of My in the physio-biochemical reactions of many plants [20], in addition to NPs that act as stimulators of soybean plant growth [86]. These findings are in line with those of a previous study [87] that reported that the growth and physiological attributes markedly increased in several crop plants as a result of the use of MgONPs. Notably, many factors, such as the chemical and physical properties of soil, including its pH, texture, and organic matter content, significantly contributed to the fluctuation in the results between the MgONP_1_ and MgONP_2_ applications [88].

The enhanced impact of the use of MgONPs on the physiological and growth parameters reflects the absorption of both micro- and macronutrients. However, the maximum values for all the leaf nutrient contents, except for the LMnCs, were obtained in the plants sprayed with either MgONP_2_ or MgONP_1_ (Fig. 5 (A-E) and Fig. 6 (A-D)). In this regard, NPs have been reported to mitigate the negative influences of salinity stress. The results in [89] demonstrate that NPs significantly contribute to the regulation of the antioxidant defense and decrease oxidative damage under abiotic stress conditions, which, in turn, enhance nutrient absorption. In their very recent studies, the authors of [90–92] assessed the stimulatory role of NPs in enhancing the plant nutrient uptake and utilization efficiency through directional delivery and controlled release. In detail, two methods have been proposed to elucidate the enhanced effect of NPs in promoting the nutritional status [93]. Numerous reviews have documented that nanoparticles act as plant nutrient carriers [94, 95], delivering nutrients to various parts of the plant and thereby improving the nutrient utilization efficiency [96]. The response of plants cultivated under abiotic stress to the application of NPs has been documented [97], indicating that NP application is the most influential at alleviating the adverse influences of saline environments. Contradictory results have been reported regarding the potential effects of NPs, as their application is thought to have useful or harmful effects on plant crops depending on the physiological stage and crop species [98–100]. Although the highest HSW values were obtained in the untreated plants, the maximum SOC and TSY values were achieved in the plants treated with MgONP_2_ and in leaves nourished with MgONP_1_ during both seasons, respectively. These results likely occurred due to the positive impacts of NPs on improving the nutrient uptake, which, in turn, enhanced the growth parameters compared with those in the untreated plants, which showed a significant decline in the leaf nutrient contents, which, in turn, negatively affected the TSY and its components [101]. Very recently, the authors of [102] postulated that NPs play a vital role in enhancing the water and nutrient use efficiency. The observed decline in the LMnCs could be attributed to the micronutrient contents (LFeCs, LMnCs, and LCuCs) and their antagonistic impacts on the availability and translocation of Mn.

### Impact of the urea phosphate x magnesium oxide nanoparticle interactions on salt-stressed soybean plants

Very limited studies have been conducted on the integrative influence of nanomaterials (NMs) with conventional fertilizers. In this work, we suggested the possibility of applying MgONPs in order to decrease the amount of applied UP. However, nanoparticle materials are more efficient than conventional materials [103]. Plants’ preference for nanoparticles is due to their unique chemical and physical properties. According to previous studies, the lowest pH in the first season and the lowest LA in the second season were recorded in plants fertilized with T_40_ and T_42_, respectively. However, when the amount of P fertilizer exceeds the permissible limits, it may disrupt nutrient metabolism, accelerate respiratory activity, and deplete carbohydrate reserves [104].

The results of our study revealed that the highest macronutrient values were achieved in soybean plants growing in soil fertilized with UP_4_ and in leaves treated with MgONP_2_ (T_43_), as shown in Table 5. Herein, the application of NPs had no effect on reducing the level of applied UP, which may be due to the significant role of UP in decreasing the pH of the rhizosphere zone, which increased the absorption of nutrients and their availability for plants [87]. However, the use of NPs has a great effect in reducing the rate of applied UP, as shown in LZnC and LCuC; meanwhile, the highest values of LFeC and LMnC were obtained in plants not sprayed with MgNPs (Table 6). These results emphasize the promising impact of NP fertilizers in improving nutrient uptake depending on their distinctive properties, such as their small size, large specific surface, and ease of uptake [105]. In other words, the enhanced effect of a high level of UP may be due to the fact that under saline-sodic conditions, plants are unable to absorb large amounts of P. NFs, on the other hand, are designed to release nutrients slowly, allowing plants to uptake them more efficiently [106]. Therefore, NP fertilizers are considered a complement to conventional fertilizers, not their replacement. This assumption was confirmed by the abundant amount of clear evidence in our research. However, the maximum TSY and their related characteristics, except SOC during the second season, were associated with soybean plants treated with a high level of UP (UP_4_) and MgNPs, irrespective of their dose, such as T_42_ and T_41_ (as shown in Table 9). This result possibility occurred due to the impact of UP4 in modulating root growth and promoting the uptake of soil nutrients [58].

### The integrative effects (antagonism and synergism) between nutrients

Crop productivity evaluation depends on understanding the dynamics of the adsorption, transport, and biological interactions of nutrients [107], and maximizing plant productivity largely depends on the synergistic interactions between macro- and/or micronutrients [108]. The relation between N and P has been reported in several reviews. In their earlier studies [109, 110], documented that the availability of N can modulate the responses of P starvation, and that the addition of N activates P acquisition in phosphorus-deficient soils, similar to the results reported in Kamel’s work [111]. In this context, our results emphasize the negative relationship between the P content and Fe, Zn, and Cu contents. In the research performed in [112], studied the antagonistic effect of Zn with other elements, such as Fe and Cu.

The contradictory findings among both growth seasons (2022 and 2023) indicate that undesirable physiochemical soil properties, such as salinity and/or sodicity could be the main reason for nutritional balance disorders [113]. However, the enhancement in the Fe absorption and accumulation under P-deficient conditions has been suggested as an alternative reason [114]. Furthermore, once both P and Fe are absorbed through the roots, Fe interacts with P, resulting in reduced P translocation to the leaves [115]. The same authors also observed interactions between N and Zn, with the N application improving the Zn uptake. These results confirm the synergistic effect between N and Zn.

## Conclusion

Salinity and sodicity are among the most severe constraints to soybean production, adversely affecting nutritional status, physiological attributes, yield, and its components. Proper nutrition management, including the selection of appropriate fertilizer types, optimal application rates, and precise timing, is a key strategy to maximize productivity under such stress conditions. This research reveals that soil application of UP combined with foliar spraying of MgONP offers a promising approach to mitigate the adverse effects of saline-sodic soils. Our results indicate that UP_4_ had the greatest impact on certain leaf nutrient contents (N, P, K, Ca, Mg, Mn, and Zn), HSW, and TSY, although the best growth and physiological attributes were obtained with UP_3_. Simultaneously, foliar application of MgONP significantly improved most studied traits compared with untreated plants. Considering the individual effects, the combination of either UP_3_ or UP_4_ with MgONP_1_ or MgONP_2_ produced the most favorable outcomes under saline–sodic soil conditions.

## Data Availability

The datasets used and analyzed during the current study available from the corresponding author on reasonable request.
